# Evidence of local adaptation in a waterfall-climbing Hawaiian goby fish derived from coupled biophysical modeling of larval dispersal and post-settlement selection

**DOI:** 10.1186/s12862-019-1413-4

**Published:** 2019-04-11

**Authors:** Kristine N. Moody, Johanna L. K. Wren, Donald R. Kobayashi, Michael J. Blum, Margaret B. Ptacek, Richard W. Blob, Robert J. Toonen, Heiko L. Schoenfuss, Michael J. Childress

**Affiliations:** 1 Department of Ecology and Evolutionary Biology, University of Tennessee Knoxville, Knoxville, TN 37996 USA; 2 The ByWater Institute, Tulane University, New Orleans, LA 70118 USA; 3 Department of Biological Sciences, Clemson University, Clemson, SC 29634 USA; 4 Department of Oceanography, School of Ocean and Earth Science and Technology (SOEST), University of Hawai‘i at Mānoa, Honolulu, HI 96822 USA; 5 Joint Institute of Marine and Atmospheric Research, University of Hawai‘i at Mānoa, Honolulu, HI 96822 USA; 6 Pacific Islands Fisheries Science Center, NOAA/NMFS, NOAA IRC, Honolulu, HI 96818 USA; 7 Hawai‘i Institute of Marine Biology, University of Hawai‘i at Mānoa, Kāne‘ohe, HI 96744 USA; 8 Aquatic Toxicology Laboratory, St. Cloud State University, St Cloud, MN 56301 USA

**Keywords:** larval transport, morphology, oceanography, individual-based models, Hawai‘i

## Abstract

**Background:**

Local adaptation of marine and diadromous species is thought to be a product of larval dispersal, settlement mortality, and differential reproductive success, particularly in heterogeneous post-settlement habitats. We evaluated this premise with an oceanographic passive larval dispersal model coupled with individual-based models of post-settlement selection and reproduction to infer conditions that underlie local adaptation in *Sicyopterus stimpsoni*, an amphidromous Hawaiian goby known for its ability to climb waterfalls.

**Results:**

Our model results demonstrated that larval dispersal is spatio-temporally asymmetric, with more larvae dispersed from the southeast (the Big Island) to northwest (Kaua‘i) along the archipelago, reflecting prevailing conditions such as El Niño/La Niña oscillations. Yet connectivity is nonetheless sufficient to result in homogenous populations across the archipelago. We also found, however, that ontogenetic shifts in habitat can give rise to adaptive morphological divergence when the strength of predation-driven post-settlement selection crosses a critical threshold. Notably, our simulations showed that larval dispersal is not the only factor determining the likelihood of morphological divergence. We found adaptive potential and evolutionary trajectories of *S. stimpsoni* were greater on islands with stronger environmental gradients and greater variance in larval cohort morphology due to fluctuating immigration.

**Conclusions:**

Contrary to expectation, these findings indicate that immigration can act in concert with selection to favor local adaptation and divergence in species with marine larval dispersal. Further development of model simulations, parameterized to reflect additional empirical estimates of abiotic and biotic factors, will help advance our understanding of the proximate and ultimate mechanisms driving adaptive evolution, population resilience, and speciation in marine-associated species.

**Electronic supplementary material:**

The online version of this article (10.1186/s12862-019-1413-4) contains supplementary material, which is available to authorized users.

## Background

The balance of migration and selection can determine the likelihood of local adaptation and evolutionary divergence within species [1–4]. For instance, migration can result in populations failing to reach local fitness optima for adaptive traits, even when natural selection favors local adaptation, such as when species occupy heterogeneous habitats [5, 6]. The theory underlying outcomes like this has been well studied, often with deterministic models of genetic architecture [7–10]. However, few natural systems have been examined in which modeled parameters are well constrained by empirical estimates of gene flow and the strength and direction of natural selection [11, 12]. There is a particular deficit in understanding the conditions that favor evolution of local adaptation in oceanic and migratory species with marine dispersing larvae [13–17]. Most prior work has focused on estimates of larval dispersal [1, 18–20] rather than integrative analysis of connectivity, post-settlement selection, and survival to reproduction [15, 21, but see 22].

Understanding how interactions between migration and selection influence the evolution of species with marine-dispersing larvae is challenging because the processes that govern population connectivity have not been well quantified. For example, a number of oceanographic transport models [23, 24] suggest that species with a longer pelagic larval duration (PLD) should have more “open” populations, whereas species with a shorter PLD should have more “closed” populations [25, 26]. Yet PLDs are often unknown. Moreover, recent studies using advanced modeling methods have not supported this previously prevailing theory. For example, studies of species with well constrained PLDs (e.g., via otolith microchemistry analyses) indicate that transport models tend to overestimate dispersal distance [27–30]. Recent comparisons of dispersal models with empirical larval drift surveys also indicate that PLDs above a critical value do not influence population connectivity [31]. In addition to oceanographic features (e.g., eddies, currents, tides), organismal attributes (e.g., larval swimming behavior, vertical migration) can impede dispersal and thus govern connectivity [19, 32–36]. Incongruent estimates of connectivity may also be due to post-settlement selection, which can mediate demographic contributions to larval pools (i.e., individuals that reach settlement sites must survive to reproduction in order to contribute to larval pools). Thus post-settlement selection and differential reproduction arising from habitat heterogeneity can potentially shape connectivity [22, 37–40] and influence evolutionary trajectories of species with marine larval dispersal [21].

Spatio-temporal variability is a well-recognized underlying aspect of population connectivity via marine larval dispersal. Not only can larval dispersal vary over space and time [41–44], so can selective pressures [45–47]. For instance, selection can vary ontogenetically if life history stages occur in different habitats (e.g., oceanic versus stream environments). And, though most analyses of connectivity assume that landscape features are deterministically static over ecological timescales [48, 49], shifts in physiographic features can generate further changes in larval dispersal and post-settlement selection [50–53]. This is well illustrated by predictions that connectivity may be altered as a consequence of climate-driven shifts in ocean currents [54–56]. Few attempts have so far been made, however, to determine whether spatio-temporal variability promulgates or constrains the likelihood of local adaptation in species with marine dispersing larvae [57–59].

Empirically constrained, coupled modeling of larval transport and post-settlement selection can be an informative approach for assessing the likelihood of local adaptation in species with marine larval dispersal. Advection-diffusion (AD) oceanographic transport models, which allow for directional or stochastic spatial-temporal variation in larval dispersal [19, 60], generate connectivity matrices that can inform spatially-explicit, individual-based models (IBMs) of post-settlement selection and reproduction [61–63]. Reciprocally, by following the fate of individuals and entire populations over time [64, 65], IBMs can inform larval transport models by describing variation in larval pool contributions arising from post-settlement selection. Coupled AD-IBM modeling can thus be particularly useful for tracking outcomes of migration and selection across life history stages that occur in different habitats or ecosystems.

Here we present the results of a spatially explicit AD-IBM model developed to examine how migration and selection shape population connectivity and local adaptation in *Sicyopterus stimpsoni*, an amphidromous fish endemic to the Hawaiian Islands known for its ability to climb waterfalls. We linked a modified Lagrangian transport model of inshore and offshore oceanographic processes for the Hawaiian Islands to IBMs for the islands of Hawai‘i (hereafter referred to as the Big Island), O‘ahu, and Kaua‘i. We utilized the AD-IBM model to assess whether natural selection is sufficient to yield morphological divergence between subpopulations that are connected via marine larval dispersal. The model was run to test the following scenarios: (1) in a closed system (i.e., local reproduction, no immigration), stream topography and discharge yield morphologically divergent subpopulation among the islands; (2) the rate and direction of morphological divergence will change by altering the strength of post-settlement selection of predation evasion and/or climbing performance; (3) alternatively, subpopulations across the archipelago are morphologically homogenous in an open system with no local reproduction (i.e., immigration only); and (4) natural selection will counterbalance high, yet stochastic larval dispersal due to variation in the strength of post-settlement selection or immigration. We then compared our modeling results to empirical estimates of the strength of post-settlement selection [66–68] and observed morphological differentiation [53] to determine the relative influences of larval dispersal and selection shaping empirical patterns of morphological divergence among populations of *S. stimpsoni* juveniles and adults.

## Results

### Oceanographic simulations of passive larval dispersal

**Fig. 1:**
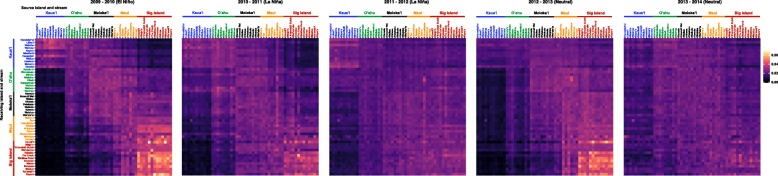
Passive larval dispersal connectivity matrices from May 2^nd^ 2009 to March 31^st^ 2014. The values in each cell are the rearward settlement probabilities for each receiving stream and the corresponding island. Each panel represents a total of 365 days, which coincides with breaks in the El Niño Southern Oscillation during the aforementioned time period. High values (yellow-orange) indicate high connectivity between streams (islands) and low values (dark purple-black) indicate low connectivity between streams (islands)

Passive larval dispersal was not spatially or temporally uniform (Figure 1). In strong El Niño years (mid 2009-mid 2010), dispersal was asymmetric with more larvae dispersed from the southeast to the northwest (i.e., from the Big Island to Kaua‘i). This asymmetry was less pronounced in strong La Niña years (mid 2010-mid 2012). The extent of asymmetry fluctuated during neutral years (mid 2012-early 2014) of the El Niño Southern Oscillation (ENSO) cycle, with an onset of asymmetry (2012-2013) followed by symmetric dispersal (2013-2014), which might have been driven by a brief period of El Niño conditions in mid 2012 (U.S. Department of Commerce, National Oceanographic and Atmospheric Administration, NOAA Research: https://www.esrl.noaa.gov/psd/enso/climaterisks/years/).

The proportion of successful larval settlement and source contributions varied among islands. The Big Island had the highest proportion (2.92%) of successful settlement, whereas Kaua‘i had the lowest proportion (1.4%) compared to all other islands (1.4-2.1%). Despite very low probabilities of successful larval transport and fluctuating patterns of passive larval dispersal, the highest percentage (42%) of local entrainment occurred on the Big Island. The Big Island also served as a source of larvae for all other islands, though with diminishing contributions up the southeast-northwest axis of the archipelago. The second highest level of local entrainment (24%) occurred on Kaua‘i, but unlike the Big Island, Kaua‘i contributed relatively few larvae to other islands. The lowest level of local entrainment (18%) occurred on O‘ahu, which also contributed relatively few larvae to other islands. Slightly higher self-recruitment occurred on Maui and Moloka‘i (23% and 21%, respectively), which contributed a similar number of larvae to other islands (16-23%).

### Linking larval dispersal with post-settlement selection

#### Scenario 1: Isolation without post-settlement selection

**Fig. 2:**
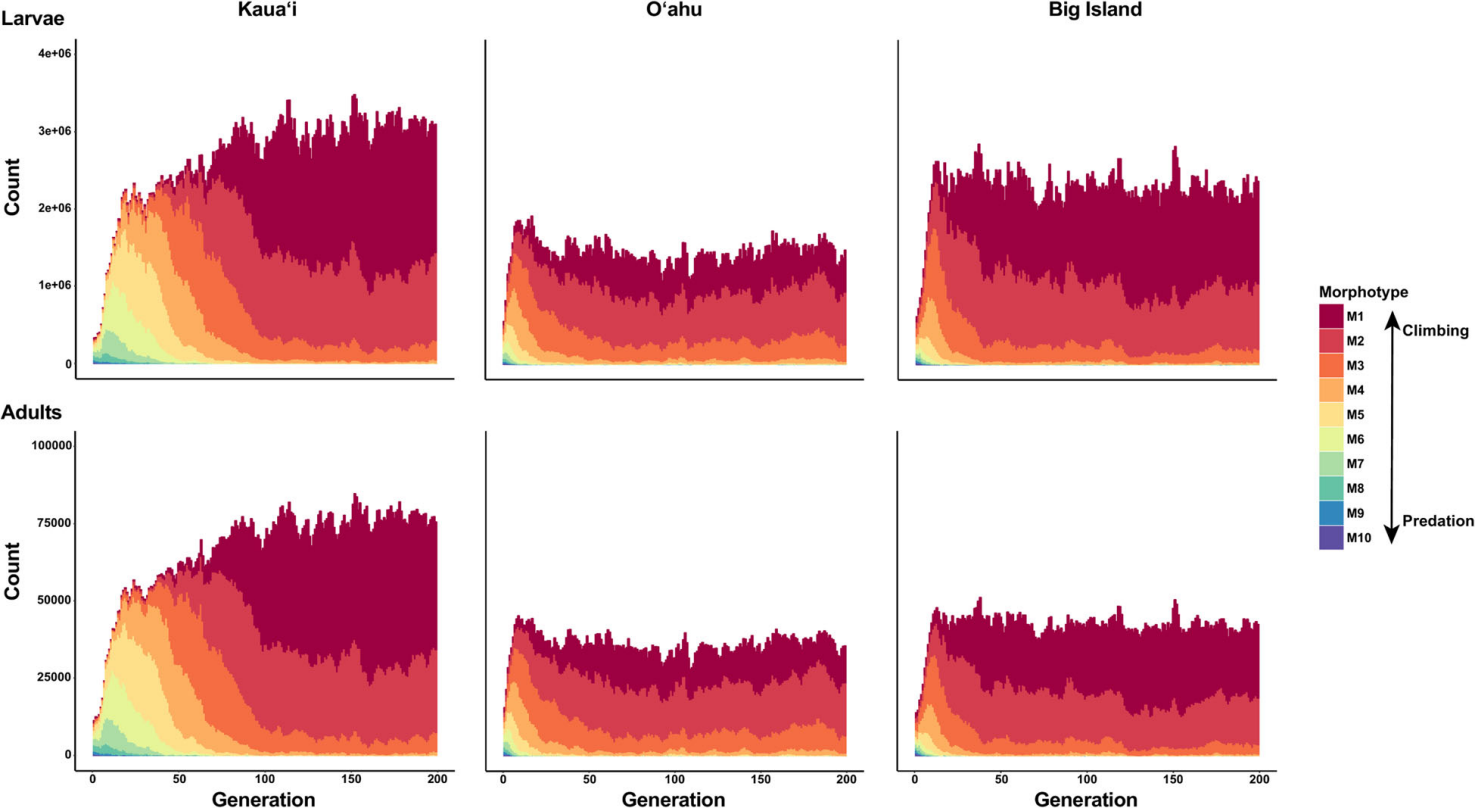
Simulated counts of larval and adult morphotypes for 200 generations on the islands of Kaua‘i, O‘ahu, and the Big Island from the individual-based models of isolation without post-settlement selection (scenario 1). Warm colors represent climbing morphotypes (M1-M4) and cool colors represent predation evasion morphotypes (M7-M10)

Differences in topographic structure and corresponding differences in stream flow did not give rise to divergent morphotypes across the archipelago. Rather, climbing morphotypes evolved and remained prevalent across all islands (Figure 2).

#### Scenario 2: Isolation with post-settlement selection

**Fig. 3:**
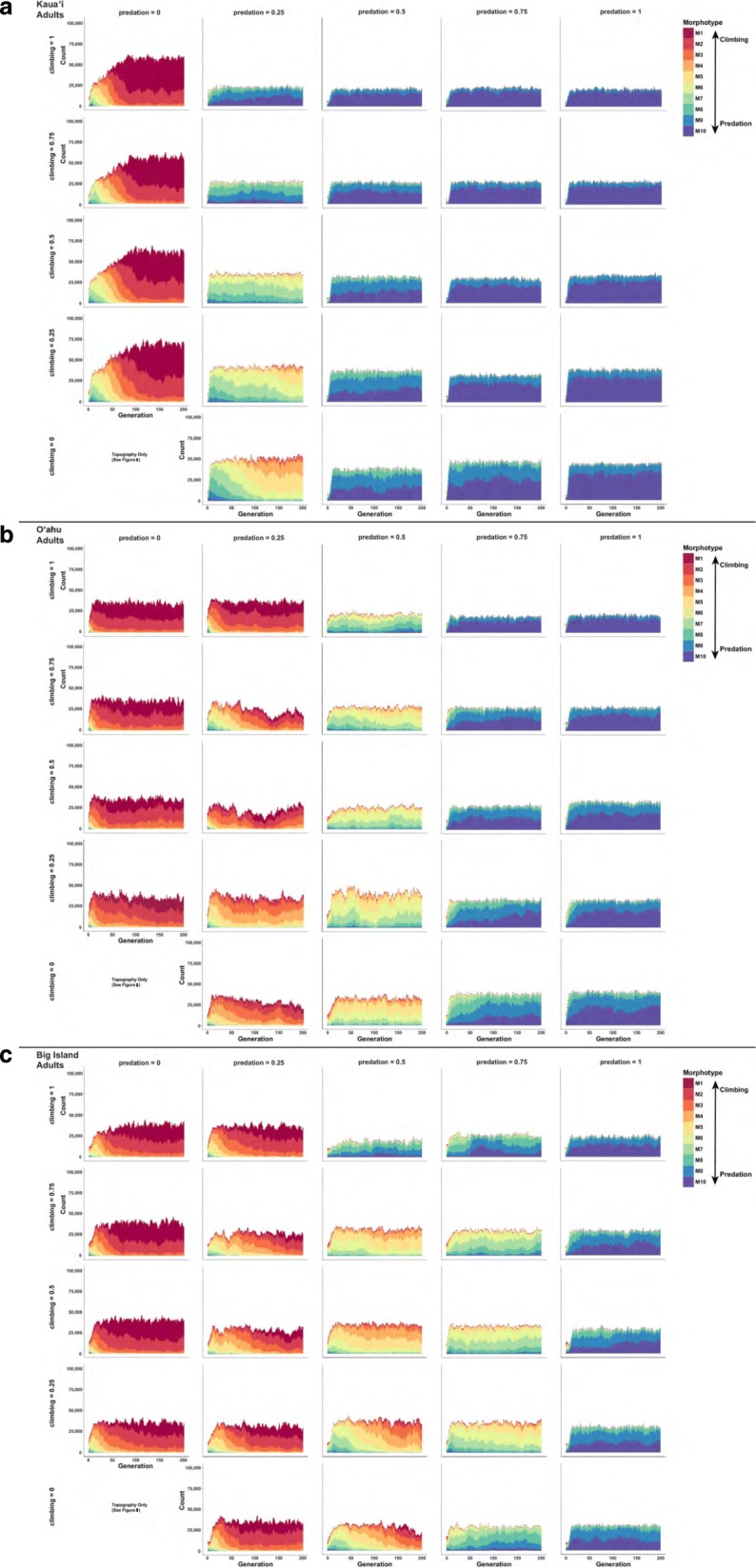
Simulated counts of adult morphotypes for 200 generations on the islands of Kaua‘i (**a**), O‘ahu (**b**), and the Big Island (**c**) from the individual-based models of isolation with varying levels (0 to 1) of post-settlement selection of predation (columns) and climbing (rows) (scenario 2). Warm colors represent climbing morphotypes (M1-M4) and cool colors represent predation evasion morphotypes (M7-M10)

When post-settlement selection was allowed to act in conjunction with isolation, morphological divergence arose across islands in accordance with predictions: predation evasion morphologies arose on Kaua‘i, whereas climbing morphologies arose on the Big Island. While morphological divergence was driven by the strength of selection from predation, the amount necessary for the evolution of predation evasion morphotypes varied across islands (Figure 3). Adult morphology does not diverge from larval morphology with increasing selection probabilities, as would be expected in isolation (i.e., larvae are products of only local reproduction and adults are products of that larval composition) (Figure 3 & Additional File 1: Figure S1).

The highest selection differentials occurred on Kaua‘i whereas the lowest occurred on the Big Island (Table 1), with a range of <0.0001 to 0.3909 across all islands. Selection differentials significantly differed between stages (Table 2). With the exception of O‘ahu, where the transition between Stage 9-10 exhibited the highest maximum selection coefficient, the largest maximum and average values of selection differentials on other islands occurred between the transition from Stage 3-4 (i.e., the post-settlement stage; Tables 1 & 2). Stage was the only significant predictor of mean selection differentials on the Big Island, whereas stage and predation probability were significant predictors of mean selection differentials on Kaua‘i. On O‘ahu, all variables were significant predictors of selection differentials.

**Table 1:** Selection differentials. Selection differentials calculated from the post-settlement selection IBMs for each island without immigration (scenario 2) and with immigration (scenario 4)

Island	Metric	Stage change								
Without immigration (scenario 2)		1-2	2-3	3-4	4-5	5-6	6-7	7-8	8-9	9-10
Kaua‘i	min	<0.0001	<0.0001	<0.0001	<0.0001	<0.0001	<0.0001	<0.0001	<0.0001	<0.0001
	max	0.14	0.29	0.39	0.31	0.30	0.31	0.29	0.26	0.29
	average	0.01	0.01	0.01	0.01	0.01	0.01	0.01	0.01	0.01
O‘ahu	min	<0.0001	<0.0001	<0.0001	<0.0001	<0.0001	<0.0001	<0.0001	<0.0001	<0.0001
	max	0.10	0.23	0.25	0.25	0.25	0.22	0.23	0.29	0.26
	average	0.01	0.01	0.01	0.01	0.01	0.01	0.01	0.01	0.01
Big Island	min	<0.0001	<0.0001	<0.0001	<0.0001	<0.0001	<0.0001	<0.0001	<0.0001	<0.0001
	max	0.10	0.28	0.36	0.28	0.30	0.32	0.24	0.21	0.21
	average	0.01	0.01	0.01	0.01	0.01	0.01	0.01	0.01	0.01
With immigration (scenario 4)										
Kaua‘i	min	<0.0001	<0.0001	<0.0001	<0.0001	<0.0001	<0.0001	<0.0001	<0.0001	<0.0001
	max	0.09	0.43	0.56	0.38	0.38	0.36	0.33	0.29	0.30
	mean	0.01	0.04	0.03	0.03	0.03	0.03	0.03	0.03	0.03
O‘ahu	min	<0.0001	<0.0001	<0.0001	<0.0001	<0.0001	<0.0001	<0.0001	<0.0001	<0.0001
	max	0.11	0.24	0.32	0.30	0.25	0.27	0.30	0.26	0.36
	average	0.01	0.04	0.04	0.03	0.03	0.03	0.03	0.03	0.03
Big Island	min	<0.0001	<0.0001	<0.0001	<0.0001	<0.0001	<0.0001	<0.0001	<0.0001	<0.0001
	max	0.47	0.50	0.53	0.41	0.34	0.34	0.37	0.36	0.25
	average	0.01	0.02	0.03	0.02	0.02	0.02	0.02	0.02	0.02

**Table 2:** Generalized linear models of selection coefficients. GLMs of selection coefficients for post-settlement selection IBMs for each island (Kaua‘i, O‘ahu, and the Big Island) without immigration (scenario 2) and with immigration (scenario 4).

Island	Parameter	Coefficient	t-value	*P-value*
Without immigration (scenario 2)				
Kaua‘i	stage	0.0005	9.39	< 0.0001
	predation probability	-0.0047	-5.59	< 0.0001
	climbing probability	-0.0009	-1.17	0.24
	predation probability × climbing probability	0.0018	1.33	0.18
O‘ahu	stage	0.0007	7.06	< 0.0001
	predation probability	-0.006	-4.19	< 0.0001
	climbing probability	-0.004	-2.84	0.005
	predation probability × climbing probability	0.004	2.09	0.038
Big Island	stage	0.0006	6.81	< 0.0001
	predation probability	0.0009	0.68	0.50
	climbing probability	-0.0001	-0.09	0.93
	predation probability × climbing probability	0.0001	0.03	0.97
With immigration (scenario 4)				
Kaua‘i	stage	0.0012	4.61	< 0.0001
	immigration rate	-0.0105	-2.46	0.01
	predation probability	0.0022	0.46	0.65
	immigration rate × predation probability	0.0289	4.13	< 0.0001
O‘ahu	stage	0.0015	6.63	< 0.0001
	immigration rate	0.0053	1.49	0.14
	predation probability	0.0193	4.90	< 0.0001
	immigration rate × predation probability	-0.0038	-0.66	0.51
Big Island	stage	0.0008	5.24	< 0.0001
	immigration rate	-0.0003	-0.12	0.91
	predation probability	0.069	2.37	0.02
	immigration rate × predation probability	0.0013	0.31	0.76

The RDAs explained 65-89% of the total variance in morphotype evolution across all islands. Morphotype evolution was significantly correlated with year, predation, climbing, and the interaction between predation and climbing (Table 3). However, each parameter explained <1.52% of the variance in morphotype evolution with the exception of predation (Table 3), which explained 16.10% of the observed morphotype variance on Kaua‘i, 19.96% on O‘ahu, and 24.42% on the Big Island.

**Table 3:** Redundancy analysis (RDA) models of morphological evolution. RDAs of morphological evolution for post-settlement selection IBMs for each island without immigration (scenario 2) and with immigration (scenario 4). * *P* < 0.0001

Island	R^2^_adj_	F_(4,250867)_	Model parameter	Coefficient	Variance partitioned (%)
Without immigration (scenario 2)					
Kaua‘i	0.65	113964*	year	-0.99	0.63*
			predation selection	0.99	16.10*
			climbing selection	-0.01	0.06*
			predation selection × climbing selection	0.67	0.02*
O‘ahu	0.89	553293*	year	-0.02	0.06*
			predation selection	0.99	19.96*
			climbing selection	-0.04	0.04*
			predation selection × climbing selection	0.66	0.23*
Big Island	0.87	407005*	year	-0.13	1.52*
			predation selection	0.98	24.42*
			climbing selection	0.64	0.86*
			predation selection × climbing selection	0.67	0.05*
With immigration (scenario 4)					
Kaua‘i	0.94	896157*	year	-0.01	< 0.001 *
			immigration rate	-0.23	0.02 *
			predation selection	0.96	25.93*
			immigration rate × predation selection	0.60	1.93*
O‘ahu	0.92	587798*	year	-0.02	< 0.001*
			immigration rate	-0.29	1.37*
			predation selection	0.89	43.23*
			immigration rate × predation selection	0.43	11.50*
Big Island	0.78	187930*	year	-0.04	< 0.001 *
			immigration rate	-0.11	2.90*
			predation selection	0.95	33.28*
			immigration rate × predation selection	0.59	6.57*

#### Scenario 3: Immigration without post-settlement selection

**Fig. 4:**
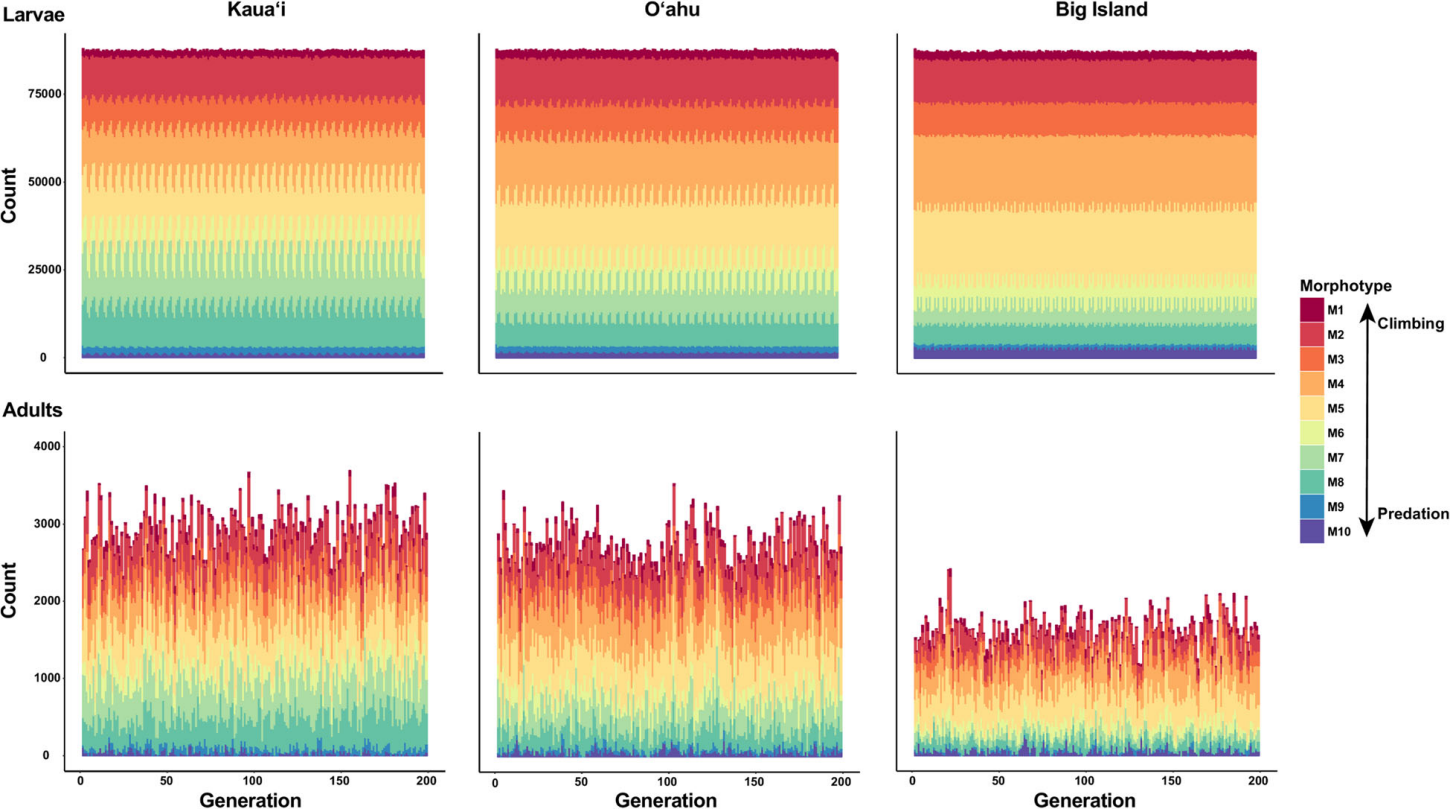
Simulated counts of larval and adult morphotypes for 200 generations on the islands of Kaua‘i, O‘ahu, and the Big Island from the individual-based models of immigration without post-settlement selection (scenario 3). Warm colors represent climbing morphotypes (M1-M4) and cool colors represent predation evasion morphotypes (M7-M10)

Under conditions of immigration without post-settlement selection, populations on all islands evolved morphotypes that are intermediate between climbing and predation morphologies (Figure 4). Additionally, adult morphotypes were reflective of larval morphotypes.

#### Scenario 4: Immigration with post-settlement selection

**Fig. 5:**
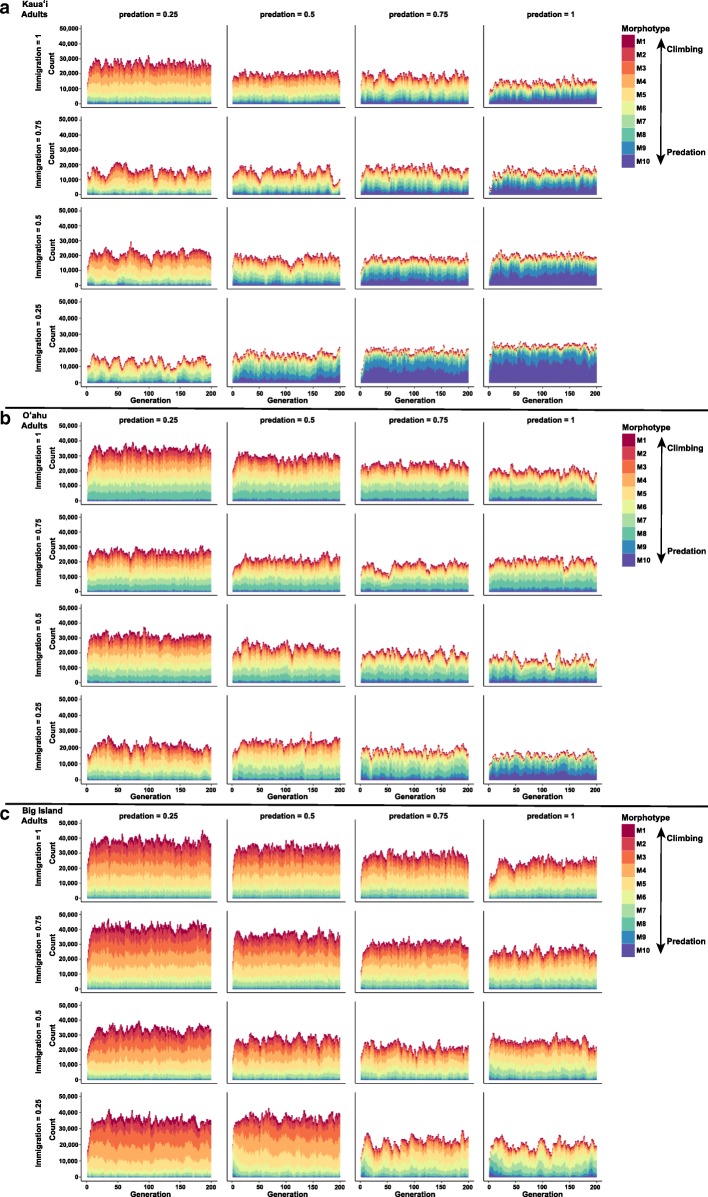
Simulated counts of adult morphotypes for 200 generations on the islands of Kaua‘i, O‘ahu, and the Big Island from the individual-based models of immigration (ranging from 25%-100%) with varying levels (0.25 to 1) of post-settlement predation selection (scenario 4). Warm colors represent climbing morphotypes (M1-M4) and cool colors represent predation evasion morphotypes (M7-M10)

When post-settlement selection was allowed to act in conjunction with immigration, morphological divergence arose across islands in accordance with predictions: predation evasion morphologies arose on Kaua‘i, whereas climbing morphologies arose on the Big Island. Morphological divergence was driven by both the strength of selection from predation and its interaction with immigration (Tables 2 & 3). The amount of predation selection necessary for the evolution of predation evasion morphotypes varied across islands (Figure 5).

Adult morphology diverged from larval morphology with increasing predation probability relative to immigration, as would be expected if post-settlement selection overcomes gene flow from immigration (Figure 5, Additional File 2: Figure S2, & Additional File 3: Figure S3).

The highest selection differentials occurred on Kaua‘i whereas the lowest occurred on the Big Island (Table 1), ranging from <0.0001 to 0.56. Selection differentials differed significantly between stages across all islands (Table 2). And with the exception of O‘ahu, where the transition between Stage 9-10 exhibited the highest maximum selection coefficient, the largest maximum and average values of selection differentials occurred during the transition between Stage 3-4 (i.e., the post-settlement stage; Table 1). Predation probability was the only other predictor of mean selection differentials on O‘ahu and the Big Island. It was not a predictor of mean selection differentials on Kaua‘i, where mean selection differentials were significantly correlated with immigration rate and the interaction between immigration rate and predation probability (Table 2).

The RDAs explained 78-94% of the total observed variance in morphotype evolution across all islands. Morphotype evolution was correlated with year, immigration, predation, and the interaction between immigration and predation (Table 3). Predation explained the highest proportion of variance in morphotype for each island (25.93% - Kaua‘i, 43.23% - O‘ahu, and 33.28% - Big Island; Table 3). The interaction between immigration and predation explained 11.50% of observed variance on O‘ahu, 6.57% on the Big Island, and only 1.93% on Kaua‘i. Immigration and year explained less than 3% and 0.01%, respectively, of morphotype variance on each island.

## Discussion

Coupled AD-IBM modeling demonstrated that the likelihood of local adaptation in a diadromous fish depends on the spatio-temporal balance of pelagic larval dispersal and post-settlement selection [12]. Our results affirm theoretical expectations that passive larval dispersal facilitated by ocean currents can result in homogenization [23, 60, 69], but we also found that dispersal asymmetries can arise due to climate-driven (i.e., ENSO) fluctuations in oceanic conditions. Our model simulations additionally showed that spatio-temporal variation in post-settlement selective pressures can override the homogenizing effects of passive larval dispersal, where differential probabilities of survival and reproduction result in adaptive evolution and morphological divergence among populations. But contrary to the expectation that post-settlement selection pressures from both predation and climbing drive population divergence, we found that predation alone is likely the primary driver of population divergence in *S. stimpsoni*. Notably, we also found that the amount of larval immigration was not a strong determining factor of morphological evolution. Rather, the strength of predation-driven, post-settlement selection and its interaction with immigration appear to shape morphological divergence across the Hawaiian archipelago, which is consistent with Fisher’s Theorem of Natural Selection [12, 70]. In other words, the strength of selection and rate of change in the mean morphotype increase as trait variance increases. In our simulations, this is driven by the continued influx of maladaptive morphotypes through immigration.

### Passive larval dispersal

The complex oceanic hydrodynamics surrounding the Hawaiian Islands makes it difficult to generalize about the dispersal of pelagic propagules across the archipelago [71]. Nonetheless, it has long been hypothesized that mesoscale eddies that form on the leeward side of the Hawaiian Islands prevent larvae from being swept away, and thus promote retention and settlement near islands [72–74]. Contrary to this idea, recent studies have found little or no association between mesoscale eddies and local larval retention in the Hawaiian Islands [75, 76], but instead facilitated larval transport to other islands [77]. Although, our AD model simulations do not directly test for the independent influences of these factors, they do indicate that the combination of mesoscale eddies and prevailing current flow patterns (southeast to northwest) contribute to local retention and larval transport among islands [34, 78, 79]. While average estimates of larval settlement were low across the Archipelago (< 3%), we found evidence of local larval retention around each island with the highest proportions of local larval retention occurring around the Big Island (42%). We also found asymmetric larval dispersal from the southeast (i.e., the Big Island) to the northwest (i.e., Kaua‘i). These results suggest that mesoscale eddies could contribute to increased larval transport among islands, which is consistent with the findings of Wren et al. (2016) [77]. However, future work should directly quantify the independent effects of mesoscale eddies from prevailing current flow patterns to understand their respective influences on *S. stimpsoni* larval dispersal.

While it is evident that larval dispersal and spatial coagulation of larval cohorts [80] vary because of dynamic nearshore and offshore oceanographic conditions [81–85], our AD modeling also illustrates the importance of considering large-scale, climatic variation in assessments of population connectivity. ENSO and other large-scale climate-driven phenomena can exhibit small temporal-scale variability that enhance or suppress larval dispersal [85–89]. Consistent with evidence of ENSO influencing population connectivity of ocean-dwelling species across the Hawaiian archipelago and elsewhere in the Pacific [19, 90], we found that ENSO likely influences *S. stimpsoni* larval dispersal (Figure 1). Parallel patterns of temporal and spatial fluctuations in F_ST_ estimates from microsatellite markers among subpopulations of *S. stimpsoni* [53] serve as further evidence that larval cohort aggregation and composition shift in response to ENSO cycles.

Our AD model was conservative with respect to larval behavior and life history (e.g., the model did not consider larval mortality, vertical migration, or swimming behavior). Incorporating other parameters into the model- including life history variation [36], larval behavior [79], and selection ‘at sea’ (i.e., selection against long distance dispersal, which could increase the probability of larval loss)- would likely improve estimates of larval transport, local retention and self-recruitment (i.e., natal homing [91]). In combination with persistent natural and anthropogenic perturbations in oceanic island streams [92–97], theory would predict that such conditions would result in the evolution of dispersal polymorphisms [98] to protect against extirpation and extinction. Accounting for the possibility of alternative dispersal strategies [99–101], which have been found in other diadromous species [102–108], could shift the balance between larval transport and self-recruitment estimates. Indeed, self-recruitment could be many times greater than predicted by our AD model [1, 109–111]. Accordingly, the modeling framework we have so far developed offers a basis for further investigations including, but not limited to, modeling outcomes of alternative dispersal strategies to better understand the implications of life history variation in amphidromous gobies like *S. stimpsoni* [102, 112, 113].

### Settlement site topography

We expected that differences in stream topography (e.g., slope, waterfall locations, and discharge) would result in divergent morphotypes across the archipelago, where steep-sloped streams with fast flows would harbor fish with long, shallow bodies, while shallow-sloped streams with slower flowing water have fish with short, deep bodies. To the contrary, under conditions of no immigration and selection probabilities turned off, we found that streamlined “climbing” morphotypes evolved in all populations, which were initially seeded with a homogenous distribution of morphotypes, regardless of prevailing topography (Figure 2). This finding suggests that, upon initial colonization (likely during the Pleistocene [14]), *S. stimpsoni* exhibited a streamlined body shape that better enabled it to penetrate steep-sloped watersheds across the archipelago. At that time, streams on the fully-formed main islands (Maui, Moloka‘i, O‘ahu, and Kaua‘i) were most likely steeper in slope and perhaps more homogenous in topography compared to current conditions. Thus our model results offer support for the idea that initial colonization of the archipelago by amphidromous fauna reflects temporally dynamic, yet time specific opportunities, where habitat suitability and availability shift with island age [114, 115].

### Post-settlement selection

#### Selection differentials

Our findings illustrate that strong selection from post-settlement mortality during juvenile recruitment can promote divergence [13, 116, 117] because watersheds across the Hawaiian Islands are heterogeneous. Though environmental gradients on each island are similar in scale, there are stark contrasts between islands across the archipelago - heavily eroded watersheds with high sedimentary loads and waterfalls far inland on older islands, and steep watersheds produced by recent volcanic flows with fast moving clear water and waterfalls close to shore on younger islands. Thus larvae recruiting from non-local sources may encounter highly unsuitable habitat. Prior work has shown, for example, that newly recruiting juveniles are exposed to strong directional selection favoring either climbing performance on the Big Island or predation performance on Kaua‘i [66–68, 118]. Our findings also are consistent with inferences that variation in non-linear selection between islands and watersheds can result in complex fitness surfaces that allow for the evolution of locally adapted populations [66].

IBM simulations intended to assess outcomes of post-settlement selection without immigration (scenario 2) indicated that selection is strongest during recruitment (ontogenetic Stage 3-4). This is consistent with expectations that post-settlement selection ought to be strongest during this transition stage because stream topographic structures (i.e., the proportion of reach types and presence of waterfalls) dictate that selection from predation and climbing occurs soon after stream re-entry. It is worth noting, however, that the influence of associated model parameters (e.g., predation and climbing probabilities, and the interaction of predation and climbing probabilities) on selection differentials varied for each island (Table 2).

Similarly, IBM simulations intended to assess outcomes of post-settlement selection with immigration (scenario 4) indicated that selection is strongest during recruitment (ontogenetic Stage 3-4). But, in contrast to simulations without immigration (scenario 2), allowing for immigration resulted in selection differentials being strongly correlated with either predation probability (O‘ahu and the Big Island) or interactions between immigration and predation probability (Kaua‘i). Notably, because our modeled selection differentials are congruent with empirical estimates of predation selection on *S. stimpsoni* [66, 68], this finding supports prior interpretations and inferences about the evolution of morphological divergence among populations of *S. stimpsoni* [53, 66, 68].

Our IBM selection differentials and morphotype results were pooled averages within an island, whereas our empirical estimates of the strength of selection and morphology were measured on a stream basis [66–68, 118]. Despite these differences in geographic scale, our simulation results and conclusions are comparable to our empirical findings. In natural populations, the strength of predation selection differentials on fitness-related traits (e.g., mid-body) ranged between 0.021-0.28 [66, 68]. In our simulations, once the mean of the selection differential exceeded 0.03, predation morphologies could evolve on Kaua‘i regardless of immigration rate. However, the degree of predation morphotype (i.e., 6, 7, 8, or 9) did vary with immigration rate (Figure 5). In contrast, even when the selection differentials reached or exceeded this threshold value on the Big Island, predation morphologies never evolved even at low levels of immigration, which is consistent with natural populations of *S. stimpsoni* residing in Big Island streams [66].

#### Morphological divergence

Theoretical and empirical studies have demonstrated that predators can engender adaptation and persistence of prey in a fluctuating environment [12, 119–121]. This may occur through a selective push, in which selection moves the mean of a trait towards a local optimum by predators consuming prey with lower fitness due to the trait in question. It may also occur as a result of the ‘evolutionary hydra effect’, in which predation reduces prey density and then increases prey birthrate, resulting in more selective events per unit time, which effectively reduces generation time [122]. Our results indicate that with post-settlement selection, regardless of immigration (i.e., whether it is included in the model or not, or whether the rate varied), distinct morphologies evolve on each island in accordance with prior predictions (i.e., predation evasion morphologies on Kaua‘i, climbing morphologies on the Big Island). The recovered patterns of divergence were principally driven by the strength of predation-derived selection (Table 3), which is consistent with the pattern of predation-driven divergence found across a broad range of taxa [122–129]. However, the strength of selection required for the evolution of predation evasion morphotypes varied among islands. Much weaker predation-driven selection was necessary on Kaua‘i compared to the Big Island or O‘ahu (Figure 3). This is well illustrated in model runs with immigration, which showed that predation evasion morphotypes do not evolve on the Big Island even when predation-driven selection is strong, whereas predation evasion morphotypes could evolve on Kaua‘i regardless of the immigration rate (Figure 5). This is highlighted by the finding that the interaction of predation-driven selection and immigration, not the strength of predation-driven selection alone, was the driving factor for adaptive evolution on Kaua‘i (Tables 2 & 3). These results suggest that the adaptive potential and adaptive evolutionary trajectory of *S. stimpsoni* may be greater on islands that have strong environmental gradients and that receive recruits with greater variance in morphology due to immigration (Figure 5).

Contrary to expectations derived from observations of natural populations of *S. stimpsoni* on the Big Island and Kaua‘i [66], estimates of the opportunity for selection were greater for the non-primary pressure on each island (i.e., climbing on Kaua‘i, predator evasion on the Big Island) than the primary pressures. Canonical rotations of the nonlinear gamma matrix (i.e., a canonical transformation that identifies the major axes of fitness surfaces and facilitates the detection of stabilizing or disruptive selection) demonstrated that individuals from Kaua‘i and the Big Island occupy regions near their local fitness peaks for some traits [66]. Therefore, selection for predator evasion on Kaua‘i and climbing on the Big Island may be less effective in promoting morphological change in *S. stimpsoni* than the non-primary pressures because variation of functionally important traits may have been reduced by directional or stabilizing selection. Our model simulations do not recover this relationship for two reasons. First, the modeled morphotypes are on a linear axis of 1-10, with morphotype being coded as a univariate trait. Consequently, the variance for some fitness-related traits could not be reduced by selection and non-selected traits maintain a high level of variance. This increases the potential for greater non-primary selective pressures to operate on each island (i.e., climbing on Kaua‘i and predation on the Big Island) resulting in greater opportunities for selection in those directions; however, this possibility could not be explored in our models. Second, this inconsistency may instead be reflective of much lower connectivity in nature than was accounted for in our oceanographic models, possibly due to a range of factors (e.g., differential larval mortality, larval swimming behavior, vertical migration, or local retention), which would be expected to greatly reduce variance in larval cohort morphology through cohort coagulation [52, 53]. Resolving this incongruence thus warrants continued exploration, parameterization, and quantification of spatio-temporally fluctuating processes that contribute to population connectivity in marine and diadromous species with marine dispersing larvae.

### Connectivity and conservation

Further work on population connectivity also stands to advance understanding of population resilience in an ever changing, human-dominated environment. This is particularly pertinent to species endemic to oceanic islands, like the Hawaiian Islands, where escalating growth of the resident human population is imposing ever-greater pressures (e.g., via habitat alteration, pollution, resource divergence) and threats (e.g., increasing temperatures, climate-driven shifts in oceanic currents, invasive species, etc.) [56, 130–132]. Many of these concerns are evident across the Hawaiian archipelago, especially on the island of O‘ahu. Home to over 80% of the human population in the Hawaiian Islands, O‘ahu has undergone extensive urbanization over the past century [133, 134]. Populations of *S. stimpsoni* on O‘ahu are either locally extirpated (i.e., within particular watersheds) or significantly smaller than those found on other islands [92], which reflects a sensitivity to perturbation and habitat modification [97, 135]. While our model results indicate that *S. stimpsoni* larvae can recruit and that local adaptation can evolve on O‘ahu, prevailing conditions appear to be overwhelming both processes on the island. It is possible, however, that the extirpation and decline of *S. stimpsoni* on O‘ahu is a consequence of significantly lower larval dispersal than what we have modeled, exacerbated by small adult populations producing relatively few larvae. It is also possible that larval dispersal is sufficient to ‘seed’ watersheds across O‘ahu, but that in-stream conditions are so unsuitable for *S. stimpsoni* that post-settlement mortality is too high for local populations to persist. Under either set of conditions, resident populations likely cannot be “rescued” by immigration. Further modeling that accounts for additional abiotic and biotic parameters [136] could improve understanding of why *S. stimpsoni* (and other endemic amphidromous species) are nearly extirpated on O‘ahu and thus help identify strategies to best reverse losses and prevent losses on other islands in the archipelago.

## Conclusion

Unlike the terrestrial systems in the Hawaiian Islands, which exemplify adaptive radiations [137–139], the endemic amphidromous fishes, as well as the marine biodiversity of Hawai‘i, are not the products of evolutionary radiations [140–143], but see [144]. Even though the Hawaiian gobies reside within the islands like honeycreepers, *Drosophila*, and silverswords, these gobies are constrained by their phylogenetic history, which includes a life cycle that dictates oceanic larval development resulting in high gene flow amongst populations [53, 114, 135, 145]. It is this contrast between adaptive radiations amongst terrestrial species but not marine species, and the underlying evolutionary mechanisms of adaptation and speciation, for which amphidromous gobies provide distinct insight. Our study demonstrates that despite strong local adaptation resulting from the complex interplay between ocean currents, dispersal strategies, and post-settlement selection, the opportunity for ecological speciation amongst marine-associated organisms is still most likely constrained as a result of gene flow. However, with recent advances in technological and bioinformatic methodologies, phylogeographic and population genetic studies of anadromous and marine species are increasingly finding evidence that barriers to gene flow exist at fine spatial and temporal scales [22, 52, 146–155]. In combination with further development of oceanographic model simulations, especially ones parameterized to reflect empirical estimates of abiotic and biotic factors (e.g., dispersal potential, larval mortality and swimming behavior, as well as post-settlement selection and ecological conditions), such studies can advance our understanding of adaptive evolution, speciation, and population resilience in an ever-changing aquatic environment.

## Methods

### Study system

The amphidromous Hawaiian goby fish, *Sicyopterus stimpsoni*, is well suited for studying how migration and selection influence the evolution of species with marine-dispersing larvae. While adult subpopulations of *S. stimpsoni* predominantly reside in upper elevation stream habitat, dispersal among streams occurs via oceanic transport of pelagic larvae [156, 157]. Dispersal distances and among-watershed connectivity are not well known, however, as it is unclear how inshore and offshore oceanic processes affect the spatio-temporal structure of larval dispersal. It is known that recruitment to streams results from pelagic larvae cueing on freshwater plumes [158], and evidence of life history variation in another goby endemic to the Hawaiian Islands [102] suggests that the probability of post-larvae returning to natal streams may reflect a combination of active (e.g., habitat use, larval swimming behaviors) and passive (e.g., oceanographic transport) factors. Thus, it is likely that cohorts that recruit back to streams and migrate upstream to adult spawning habitat are composed of individuals originating from a combination of sources (i.e., natal and other streams). Consistent with this, patterns of little to no neutral genetic differentiation in microsatellite and mitochondrial markers, among adult subpopulations across the Hawaiian archipelago, suggest that recruiting cohorts draw from well-mixed larval pools [53, 112, 114, 135, 145, 159, 160].

During upstream migration, both predation and waterfall climbing produce brief, but intensive, episodes of selection on juvenile *S. stimpsoni* [66–68]. Juvenile recruits must escape predation (e.g., by the endemic, piscivorous sleeper, *Eleotris sandwicensis*), and individuals who survive predation face the additional selective pressure of climbing waterfalls before reaching predator-free adult habitats where *S. stimpsoni* grow, mature, and reproduce [161, 162]. Both forms of selection may lead to local adaptation in morphology among streams if additive genetic variation exists for shape differences [53, 66–68].

The strength of natural selection from predation and waterfall climbing varies according to watershed topography. The geomorphology of Hawaiian watersheds spans a topographic gradient that tracks the progression of erosion with island age [163]. Variation in topography translates to differences in the steepness of stream slope, stream depth and breadth, and surface flow rates [53, 92]. For example, streams on the Big Island are characteristically steep-sloped, and often terminate in waterfalls with little to no estuarine habitat. Individuals recruiting to “Big Island-like” streams must climb waterfalls within a few days of settlement. Therefore, natural selection should favor individuals with streamlined morphologies (i.e., long, shallow bodies) for reduced drag and better waterfall-climbing performance [164, 165]. On the other hand, streams on Kaua‘i are characteristically shallow-sloping with waterfalls that are often located kilometers inland. Individuals recruiting to “Kaua‘i-like” streams thus may have to swim upstream for weeks before escaping predation by climbing waterfalls. Therefore, natural selection should favor individuals with short, deep bodies that facilitate greater thrust production for predator evasion [164–166]. Accordingly, differences in stream topography likely constitute a mosaic of selection pressures that underlies observed patterns of divergence in body shape among sub-populations [53, 66–68] under conditions of high gene flow.

### Modeling passive larval dispersal: oceanographic simulations

Patterns of larval dispersal can change in both space and time [41–44], therefore we used a modified version of the 2D Lagrangian transport model described in Polovina et al. (1999) [167] and Wren & Kobayashi (2016) [31] to simulate larval dispersal and recruitment. As implemented here, the transport model tracks individual larvae (i.e., particles) through physical oceanographic models from spawning to settlement, moving them through ocean velocity fields. We set the diffusivity coefficient to 250 m^2^/sec ([168], Jia pers. comm.) and we used a settlement radius of 5 km around each stream mouth. For physical flow fields we used a regional implementation of the daily Hybrid Coordinate Ocean Model (HYCOM) [169], with a K-profile parameterization (KPP) mixed layer formulation for our current solutions (http://apdrc.soest.hawaii.edu/datadoc/hycom_hawaii_0.04_kpp.php). The domain of the regional HYCOM model spans 194°E-210°E and 16°N-26°N and has a 1/25° horizontal resolution and 28 active vertical layers. This model is eddy resolving, which accurately predicts mesoscale eddies that are often present in the lee of the Hawaiian Islands [170]. For simulation runs, we depth-averaged current velocities in the top 100 m (i.e., the top 7 layers of the HYCOM), excluding the surface layer (0-5 m). We used this approach because it has been shown to best predict the position and settlement of larval reef fishes from empirical studies within the Hawaiian Islands [31] and there is no evidence from plankton and midwater trawl surveys that larval goby distributions are neustonic or epipelagic (i.e., under the sole influence of the HYCOM surface layer) [171].

**Fig. 6:**
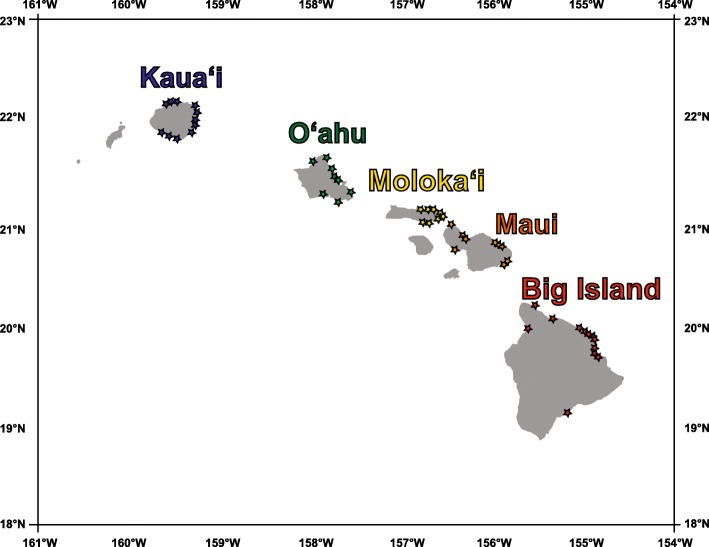
Locations of Hawaiian streams used as release/recapture points in the passive larval dispersal model

A total of 51 stream mouth locations on Kaua‘i, O‘ahu, Moloka‘i, Maui, and the Big Island were used as release and settlement sites in the model (Figure 6). One hundred virtual larvae were released daily from each stream mouth location from 2 May 2009 until 31 March 2014 (1594 days), totaling over 8 million (8,129,400) virtual larvae released during the simulation. We conducted separate preliminary AD model simulations with PLD set to 55 and 150 days post-release and found slightly but not significantly higher levels of connectivity at 150 PLD compared to 55 PLD. Further, the patterns we observed when running these preliminary simulations were very similar to the overall patterns of connectivity found when using a continuous range of PLDs. Therefore, in an effort to best represent the entire range of possible PLDs of *S. stimpsoni*, we used a continuous bimodal range of 50 days to 200 days based on estimates of age and PLD from otolith microchemistry studies [102, 172–174]. Each larva could settle as early as 50 days after release and up to 200 days after release, after which mortality occurred. In all AD simulations, larvae were assumed to be passive throughout their PLD. Simulation results were averaged across three runs (to account for stochasticity) with daily time steps.

### Linking larval dispersal with post-settlement selection: IBM development

Three independent, spatially explicit, individual-based models (IBMs) were developed and parameterized in NetLogo (6.0.3) [175] for Kaua‘i, O‘ahu, and the Big Island (respectively). We limited our IBMs to these three islands because our prior work quantified selection and morphological divergence on Kaua‘i and the Big Island [53, 66–68] allowing for direct comparisons of simulation results with empirical data. In addition, populations of *S. stimpsoni* on O‘ahu are extremely rare and perhaps locally extirpated, making this island of interest for conservation concerns. Each IBM was divided into habitat cells of 1 km^2^ and scaled to island size. The habitat cell types represented the primary habitats utilized by *S. stimpsoni*: offshore ocean and nearshore ocean, which are larvae-only cells, and estuarine and upstream, which are juvenile- and adult-only cells, according to the species life history [81, 176].

We selected eight to ten watersheds on each island representative of habitat type and elevation to include in the island-specific IBMs. Physical characteristics (length, elevation, slope) for each of the selected watersheds were determined from the Atlas of Hawaiian Watersheds and their Aquatic Resources (http://www.hawaiiwatershedatlas.com/index.html). Additional data gathered from Google Earth enabled us to develop metrics for waterfalls in the IBMs. The highest elevation areas within watersheds were excluded from the IBMs because *S. stimpsoni* do not inhabit headwater reaches [176, 177].

Patch conditions were watershed-specific with weekly discharge estimates based on historical records from United States Geological Survey stream gauges (http://hi.water.usgs.gov). Weekly discharge rates (ft^3^/sec) were averaged across all years, and an annual sine curve model was calculated for each watershed (Additional File 4: Supplemental Equations). For watersheds without gauge data (22 streams), we used log(stream channel length km), log(watershed area km^2^), and log(max elevation m) in a principal component analysis to calculate the nearest neighbor distance. Nearest neighbor distance (√((x_2_-x_1_)^2^+(y_2_-y_1_)^2^+(z_2_-z_1_)^2^)) was determined using principal component axes 1-3, and used to assign annual sine curve models of discharge rates to watershed without gauges.

Each IBM followed the survival of individual *S. stimpsoni* through ten life history stages, from late-stage pelagic larvae to adults on a weekly time step basis. Stage 1 corresponded to the final marine larval stage, with corresponding individuals having reached the nearshore waters surrounding the island but not yet having detected freshwater plumes. Thus, Stage 1 movement is randomly oriented in the offshore marine environment with respect to stream locations. Stage 2 represented the nearshore post-larval stage, during which individuals exhibit strong orientation toward freshwater plumes depending on the width and flow from each watershed. Stage 3 constituted juvenile stream fish with fully developed benthic feeding and climbing structures that orient upstream and continue to migrate until reaching suitable adult habitat and conspecific density. Stages 4 through 10 consisted of reproductively mature adult fishes that retain upstream orientation until reaching suitable adult habitat and conspecific density [176]. Each stage differentiated by growth in approximately 1 cm increments [178], which influences the probabilities of predation, growth and net reproductive output.

Each individual agent (fish) in the model possessed the following characteristics: stage (1-10), sex (male or female), position (x-y coordinates), direction (degrees), and morphotype (0-1). Morphotype consisted of a single trait on a scale from 0 to 1 that describes a continuum of body shape from long, shallow bodies (climbers) to short, deep bodies (predator evaders). Because *S. stimpsoni* morphotype heritability is unknown, we treated trait heritability as the average of the two parental morphotypes +/- 5% of the average difference between parental morphotypes, which falls within the range of narrow-sense heritability estimates for morphological traits in fishes [179, 180] and is calculated as:

1 $$ Offspring\_ morphotype=\left(\frac{\left( mother\_ morphotype\right)+ father\_ morphotye}{2}\right)+ random\left(0.05+\left(| mother\_ morphotype- father\_ morphotype|\right)\right)- random\left(0.05+\left(| mother\_ morhotype- father\_ morphotype|\right)\right) $$

Each IBM was populated with initial agents of each stage with an equal distribution of all morphotypes. Above a threshold that could cause random extinction, the initial number of agents had little effect on model output, and was therefore set at 3000 gobies to minimize that probability. Upon initiation of the model run, the IBM proceeds as a continuous loop of six subroutines executed on a weekly time step: patch conditions, movement, mortality, reproduction, growth, and immigration. Larvae (Stage 1) begin in the offshore zone of the model and undergo mortality if *natural_mortality* calculated as:

2 $$ natural\_ mortality=\left(\frac{1}{1+ stage}\right) $$

is greater than a random probability (between 0 and < 1 assigned at each time step), or move into the nearshore zone if they survive to Stage 2. Stage 2 larvae must then find a watershed inlet habitat cell in order to move into a stream or else perish. Upon entering the watershed (Stages 3-10), the probability of moving upstream depends upon the elevation gradient between the current patch and the next patch, the current hydrological discharge conditions of the patch, and the morphotype of the individual. Thus, the probability of upstream movement was calculated as:

3 $$ P(climb)=\left({\mathit{\exp}}^{- morphotype}\right)-\left(\left(1-{\mathit{\exp}}^{\left(-\left(\frac{discharge}{3}\right)+\left(\frac{depthdiff}{50}\right)\right)}\right)\ast climb\_ threshold\right) $$

where *discharge* is the logarithmic stream-specific sine curve equations (Supplemental Materials Eq. 1.1) scaled by a factor of 1/3 that converts *discharge* to a unit of climbing difficulty between 0-1. The parameter *depthdiff*, a proxy for waterfall height, is the absolute value of the elevation difference between the current patch and the next patch. *depthdiff* is scaled by a factor of 1/50 that converts this parameter to a unit of climbing difficulty, resulting in a similar distribution of climbing success as observed by Blob et al. 2008, 2010 [67, 68] and a longitudinal distribution of individuals within a stream similar to that observed in nature (Atlas of Hawaiian Watersheds and their Aquatic Resources, http://hawaiiwatershedatlas.com). The *climb_threshold* parameter is user-defined on a scale from 0 to 1, which is a primary parameter that is changed between our simulated scenarios.

Individuals that fail to climb to the next patch during the current time step remain in the same patch until the next time step or until they experience mortality. Mortality is stage-specific and can result from natural mortality (i.e., aging), predation, or competition. Natural mortality is calculated as in Equation 2. Predation mortality is calculated as:

4 $$ predation\_ mortality=\left( natural\_ mortality\ast predation\_ risk\ast \left(\left({\mathit{\exp}}^{- morphotype}\right)-0.35\right)\ast \left(\frac{1}{1+ elevation}\right)\right) $$

where *predation_risk* is user-defined on a scale from 0 to 1 as primary parameter that is changed between our simulated scenarios. An individual experiences predation mortality when *predation_mortality* is greater than a random probability between 0 and < 1 assigned at each time step. Mortality due to competition is based on carrying-capacity and is calculated as:

5 $$ competition\_ mortality=1-\left(\frac{\left( carrying\_ capacity-n\_ indivduals\_r1\right)}{carrying\_ capacity}\right) $$

where *n_individuals_r1* is the number of individuals within a 1 cell radius of an individual and *carrying_capacity* is user-defined. We set *carrying_capacity* to 100 gobies per cell, because this value resulted in an asymptotic population growth curve, whereas values below this threshold resulted in population extinction, and above this threshold resulted in exponential population growth. If competition is greater than a random probability between 0 and < 10, then an individual experiences mortality from competition.

Male *S. stimpsoni,* which are territorial, not only have an elaborate courtship ritual that precedes pair-forming and spawning but also guard fertilized egg clutches [161, 176]. Therefore only individuals of reproductive size (Stages 4-10) were allowed to reproduce if an individual of the opposite sex was within one habitat cell. Reproduction, though year-round, is also linked to stream discharge, which is seasonally variable [96]. Thus we calculated reproduction probability as:

6 $$ P\left( reproduction| discharge\right)=1.355\ast discharge-0.459\ast {discharge}^2 $$

resulting in a normal distribution of probabilities that are scaled to a unit of reproduction between 0 and 1. The number of offspring was determined by:

7 $$ offspring\_ number=\left|\left( reproduction\ast births\right)\right| $$

where *births* is a user-defined parameter ranging from 0-500, and reproduction is:

8 $$ reproduction=\left(\frac{2^{\left( stage-4\right)}}{100}\right) $$

Newly-produced larvae were assigned a morphotype as described above.

In nature, both female and male *S. stimpsoni* have indeterminate growth [95]. In the IBMs, growth rate was expressed as a logistic curve determined by stage dependent size (i.e., faster relative growth of younger, smaller individuals compared to older, larger individuals) and an annual sine curve of temperature (i.e., faster growth in warmer waters) calculated as:

9 $$ P\left( growth\left| stage\right.\right)=\left(1-\left(\frac{temperature}{29}\right)\right)+ random5<\left(\frac{age}{\left(1+{\left( stage-1\right)}^2\right)}\right) $$

where,

10 $$ temperature=\left(18.22+0.9814\ast \mathit{\sin}\left(\left(6.283\ast \left(\frac{week}{52}\right)+3.545\right)\ast \frac{180}{\pi}\right)\right) $$

and is scaled by a factor of 1/29 and added to a random number between 0 and < 5 for conversion to a unit of growth. Stage 10 individuals were determined to undergo senescence when the conditions for growth to the next stage were met, which occurred on average at ~165 weeks (i.e., 3.17 years after obtaining the size maximum).

Immigration rules in the IBMs consisted of weekly connectivity matrices from the AD model. Predicted pelagic larval morphotype distributions for these AD connectivity matrices were determined by the slope of the stream from which larvae were released, as stream slope gradient is correlated with morphotype in natural populations (i.e., individuals from steep sloping streams have long, shallow bodies, whereas, shallow sloping streams have individuals with shorter, deeper bodies [53]). The slopes of the 51 streams in the AD model were determined by regressing the distance from the mouth by the elevation of the site every 1000 meters upstream. The slope values were then used in a linear regression to predict a numerically scaled morphotype for individuals from each of the 51 streams. Predicted morphotype distributions were used to seed immigrant morphotypes (Additional File 5: Figure S4).

### Linking larval dispersal with post-settlement selection: IBM simulation scenarios

#### Scenario 1: Isolation without post-settlement selection

With age, islands progressively erode and eventually subside into the ocean [163, 181]. Thus, topographic differences of Hawaiian watersheds should shape the morphological distributions of *S. stimpsoni* such that fish on older islands (i.e., Oʻahu and Kauaʻi) have shorter, deeper bodies (e.g., a predator evader morphotype) compared to fish on younger islands (i.e., Molokaʻi, Maui and the Big Island). We therefore ran each island IBM in isolation (i.e., local reproduction only) with climbing and predation selection parameters turned off, in order to assess whether island topographic differences alone give rise to morphological divergence.

#### Scenario 2: Isolation with post-settlement selection

Similar to patterns of larval dispersal, selective pressures can change over space and time [45–47]. Because in-stream selection pressures of predation and climbing are predicted to shape morphological variation, *S. stimpsoni* in the model should evolve morphotype distributions similar to those observed in nature, consistent with biomechanical predictions of optimal shapes for predator-evasion or waterfall-climbing performance [67, 68]. Accordingly, we ran each island IBM in isolation and with varying climbing and predation post-settlement selection probabilities (incrementally from 0 to 1) to assess whether and how, post-settlement selection gives rise to morphological divergence.

#### Scenario 3: Immigration without post-settlement selection

Immigration may erode local adaptation because larval recruits from different sources may arrive at settlement sites with suboptimal morphotype distributions [12]. Natal retention might therefore be expected to increase survival and population persistence. Immigration also may be key to population persistence, however, because oceanic island streams are prone to natural and anthropogenic perturbation [92, 93]. We thus ran each island IBM with immigration only (i.e., no local reproduction) and without climbing and predation post-settlement selection to assess whether passive larval dispersal gives rise to morphological homo/heterogeneity and how immigration influences local demography.

#### Scenario 4: Immigration with post-settlement selection

Post-settlement selection may be strong enough to override the homogenizing effect of immigration via larval dispersal. We thus ran each island IBM with varying (0-1) climbing and predation post-settlement selection probabilities and varying levels of immigration, to assess the potential for morphological divergence of *S. stimpsoni* under conditions of migration and selection. The extent of immigration was set by weighting the connectivity matrices by 0.25, 0.5, 0.75, and 1, since the connectivity matrices reflect purely passive larval dispersal and thus represent a conservative estimate of dispersal across the archipelago. The weighting parameter therefore estimates the effect of reduced larval dispersal from biotic (i.e., active swimming behavior, vertical migration) or abiotic processes (i.e., temperature, salinity, chlorophyll) demonstrated in other marine and diadromous species [18] but not taken into account in our larval dispersal model. Because simulations of scenario 2 (see results) showed that climbing selection exerted negligible influence, we left climbing probability set to 1 and did not further analyze climbing for scenario 4. All IBM simulations were run 10 times for 200 generations.

### Statistical Analyses

From the oceanographic AD simulations, successful larval settlement was defined as any larvae occurring within a 5 km distance of a stream mouth. To capture inter-annual variation in larval transport success, a rearward probability connectivity matrix for each year was calculated as:

11 $$ {P}_{ij}=\frac{S_{ij}}{\sum {S}_j} $$

where *S*_*ij*_ is the number of larvae release from stream *i* (source stream) that successfully settled at stream *j* (receiving stream), and *S*_*j*_ is the total number of larvae that successfully settled at a stream regardless of release stream. Each of the cells of the *P*_*ij*_ matrices shows the probability of a particle transported to stream *j* having originated from stream *i* each year, averaged across the three simulation replicates, where each row in a matrix sums to 1. The diagonal of the probability matrices shows the amount of local entrainment for each stream, which is defined as the proportion of successfully transported larvae at each stream that originated from that same stream. We also calculated the overall proportion of local entrainment for each island across all years of the model by dividing the number of released larvae from all streams on an island that were successfully transported back to any stream on that same island.

To determine if our modeled selection parameters were within the range of empirical selection differentials [67, 68], we calculated the simulated selection differentials (*s*) for each island in tests of scenario 2 and scenario 4. Selection differentials were calculated as the difference between the mean morphotype of stage *J*_*n+1*_ minus the mean morphotype of *J*_*n*_ within each time step of the IBMs [182]. The modeled selection differential encompassed both the effects of climbing and predation selection. Consequently we used generalized linear models (GLMs) to quantify the degree to which age, predation, climbing or the interaction of predation and climbing contributed to our modeled selection differentials from IBM simulations of post-settlement without immigration (scenario 2). For IBM simulations of post-settlement selection with immigration (scenario 4), we used GLMs to quantify the degree to which age, immigration, predation, or the interaction of immigration and predation were contributing to our modeled selection differentials. Because simulations of scenario 2 showed that climbing selection exerted negligible influence on selection differentials (see results), we set the climbing probability in all IBM simulations of scenario 4 to a value of 1. Subsequently we did not use climbing as a parameter in the GLMs for scenario 4.

We used Redundancy Analysis models (RDA), a multiple linear regression ordination method [183], computed with the vegan package in R [184], to quantify the contribution of each model parameter to morphotype evolution under conditions of isolation and selection (scenario 2), as well as immigration and selection (scenario 4). We conducted independent RDAs for each island for isolation with selection, with predictor variables of year, predation, climbing, and the interaction of climbing and predation (scenario 2). For models of immigration and selection (scenario 4), we conducted independent RDAs for each island with the predictor variables of year, immigration, predation, and the interaction of immigration and predation. Because simulations of scenario 2 showed that climbing selection exerted negligible influence on morphological change (see results), we set climbing probability in all IBM simulations of scenario 4 to a value of 1. Subsequently we did not include climbing as a parameter in our RDAs for scenario 4.

We estimated the adjusted coefficient of determination (R^2^_adj_) for each model, with statistical significance determined using permutation tests to compare observed and randomized model R^2^_adj_. Since morphotypes at time *t+1* are correlated with morphotypes at time *t*, we conducted variance partitioning with partial RDAs to estimate the variance in morphotype evolution that was independently explained by each predictor variable [185, 186], thereby controlling for covariance between time points.

## Additional files

Additional File 1: **Figure S1.** Simulated counts of larval morphotypes for 200 generations on the islands of Kaua‘i (a), O‘ahu (b), and the Big Island (c) from the individuals-based models of isolation with varying levels (0 to 1) of post-settlement selection of predation (columns) and climbing (rows) (scenario 2). Warm colors represent climbing morphotypes (M1-M4) and cool colors represent predation evasion morphotypes (M7-M10). (PDF 4133 kb)

Additional File 2: **Figure S2.** Simulated counts of immigrant larval morphotypes for 200 generations on the islands of Kaua‘i (a), O‘ahu (b), and the Big Island (c) from the individual-based models of immigration (ranging from 25%-100%) with varying levels (0.25 to 1) of post-settlement predation selection (scenario 4). Warm colors represent climbing morphotypes (M1-M4) and cool colors represent predation evasion morphotypes (M7-M10). (PDF 4221 kb)

Additional File 3: **Figure S3.** Simulated counts of self-recruitment larval morphotypes for 200 generations on the islands of Kaua‘i (a), O‘ahu (b), and the Big Island (c) from the individual-based models of immigration (ranging from 25%-100%) with varying levels (0.25 to 1) of post-settlement predation selection (scenario 4). Warm colors represent climbing morphotypes (M1-M4) and cool colors represent predation evasion morphotypes (M7-M10). (PDF 4769 kb)

Additional File 4: Supplemental Equations. Sine curve equations of monthly stream discharge estimates from UGSS stream gauge data. (DOCX 101 kb)

Additional File 5: **Figure S4.** Predicted morphotype frequency distribution and island of origin of settled pelagic larvae from the dispersal. These frequency distributions were used as the inputs for pelagic larval morphology (Stage 1) in the individual-based models that included immigration (scenarios 3, and 4). (PDF 36 kb)

## Abbreviations

AD: Advection-Diffusion
AD-IBM: Advection-Diffusion-Individual Based Models
ENSO: El Niño Southern Oscillation
GLM: Generalized Linear Models
HYCOM: Hybrid Coordinate Ocean Model
IBM: Individual-Based Model
KPP: K-Profile Parameterization
NOAA: National Oceanographic and Atmospheric Administration
PLD: Pelagic Larval Duration
RDA: Redundancy Analysis
U.S.: United States
USGS: United States Geological Survey
