# Supplementary figures and images for: Evidence of local adaptation in a waterfall-climbing Hawaiian goby fish derived from coupled biophysical modeling of larval dispersal and post-settlement selection
Source: BMC Evol Biol. 2019 Apr 11;19:88. doi: 10.1186/s12862-019-1413-4 (PMC6458715; doi:10.1186/s12862-019-1413-4)

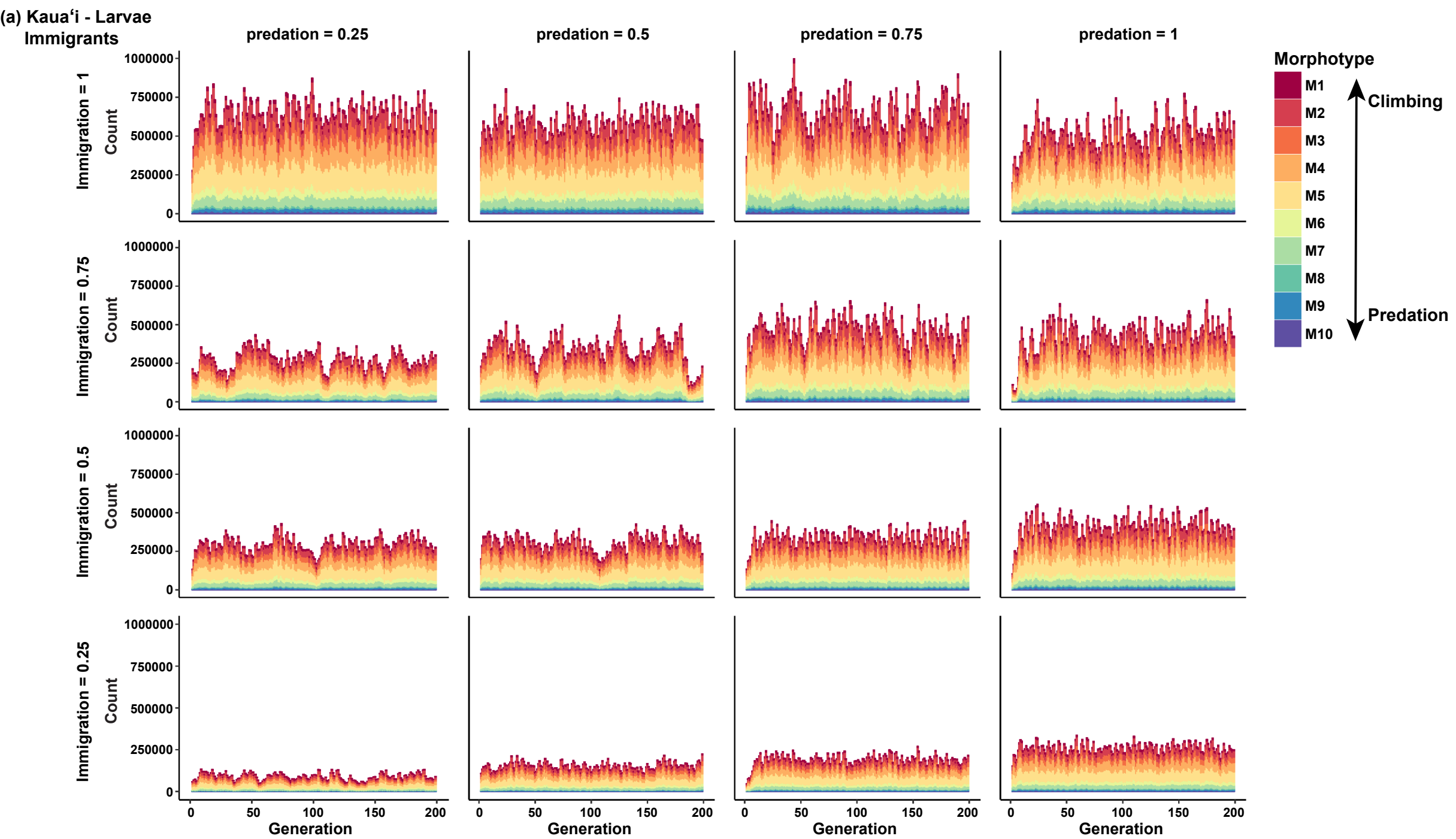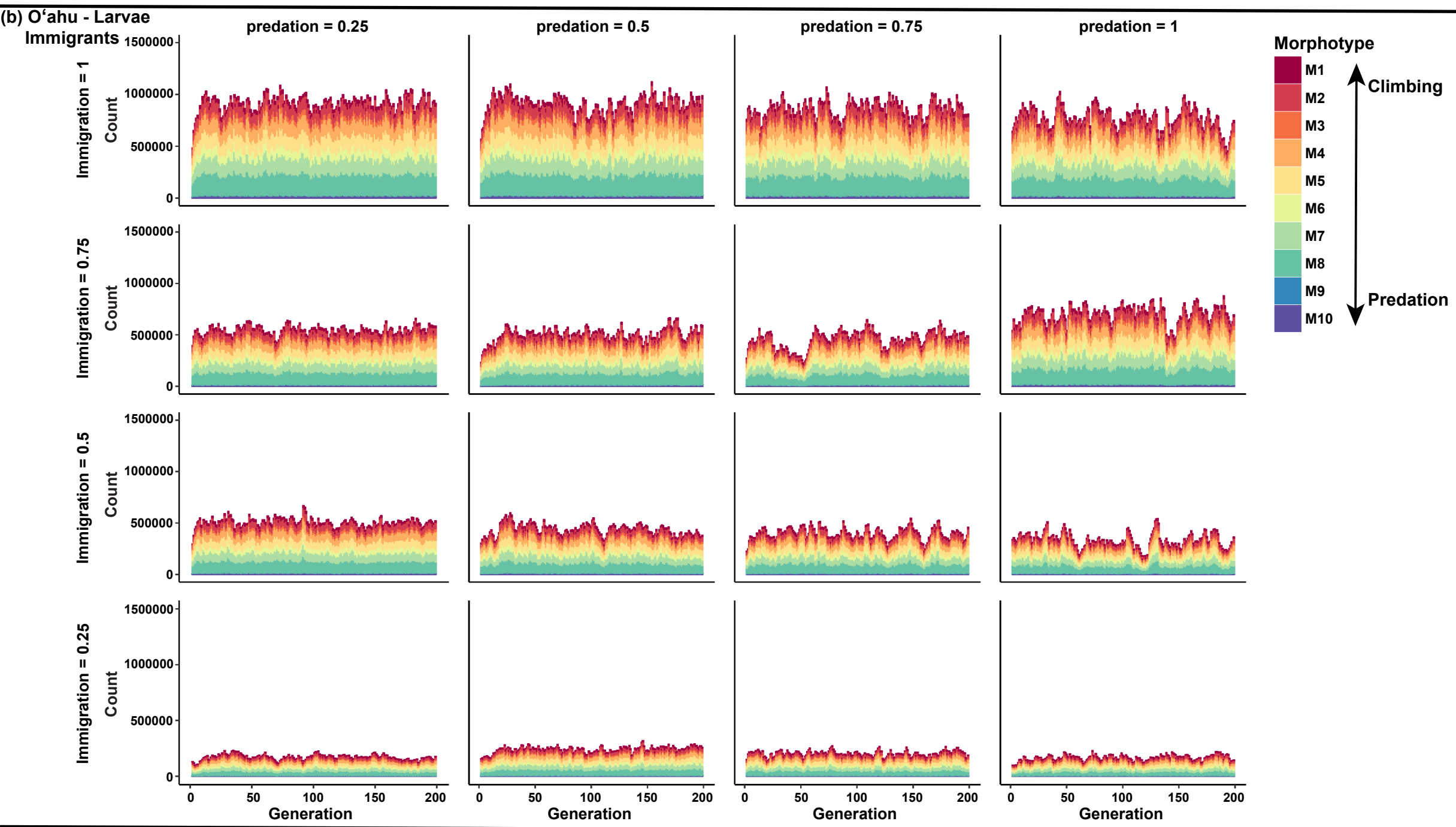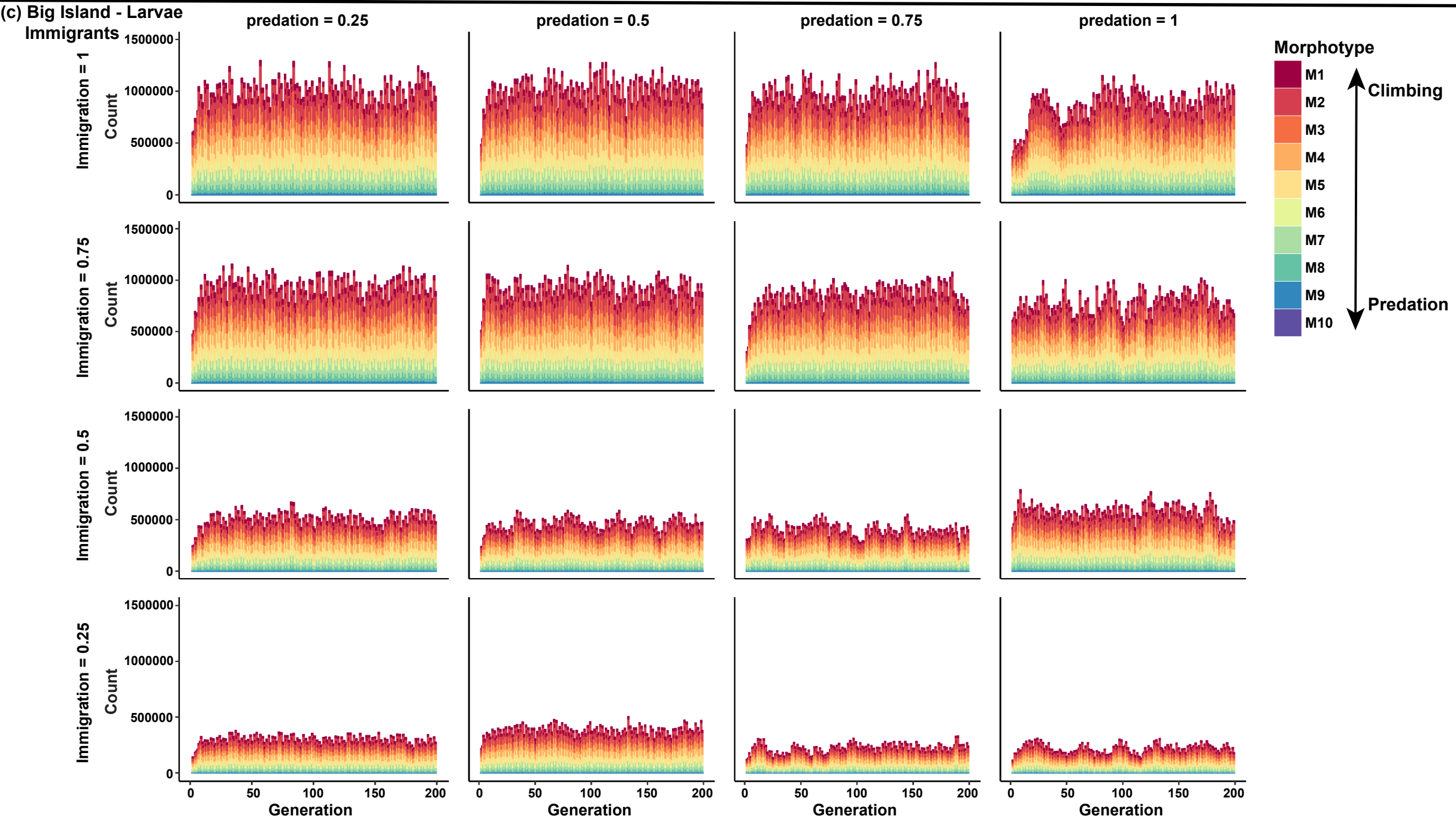

Supplement: Supplementary file 2 — Figure S2. Simulated counts of immigrant larval morphotypes for 200 generations on the islands of Kaua‘i (a), O‘ahu (b), and the Big Island (c) from the individual-based models of immigration (ranging from 25%-100%) with varying levels (0.25 to 1) of post-settlement predation selection (scenario 4). Warm colors represent climbing morphotypes (M1-M4) and cool colors represent predation evasion morphotypes (M7-M10). (PDF 4221 kb) [file 12862_2019_1413_MOESM2_ESM.pdf]

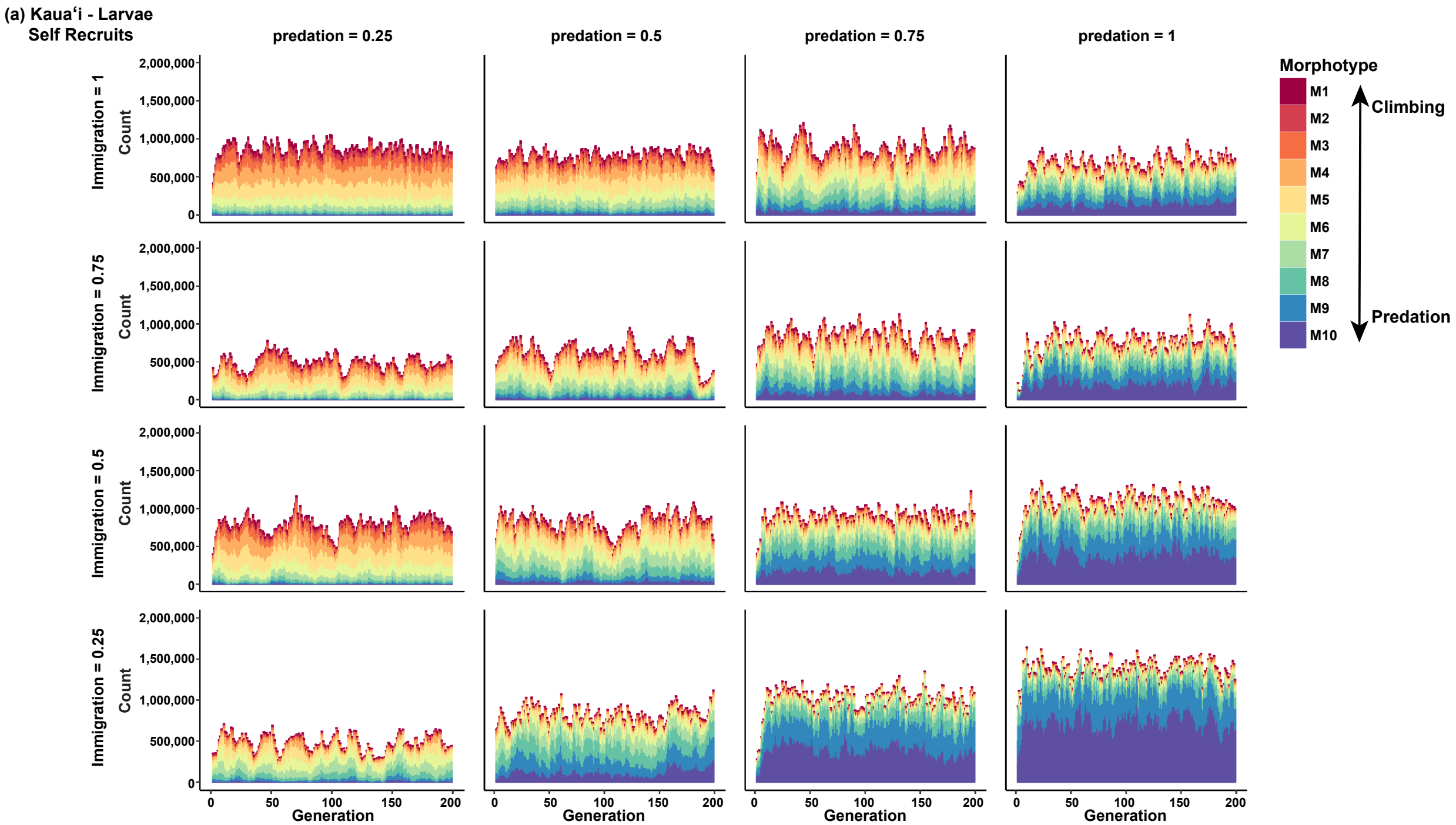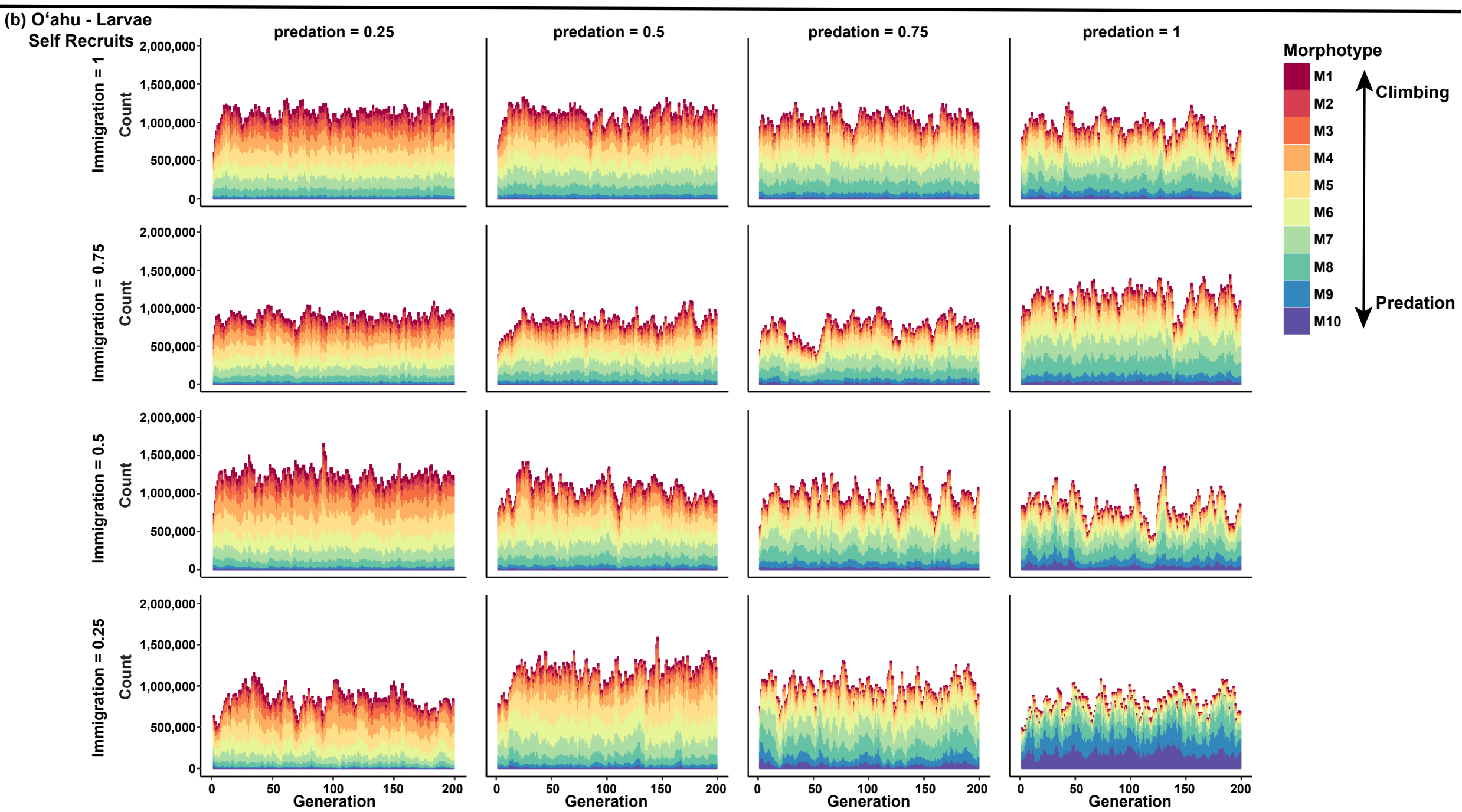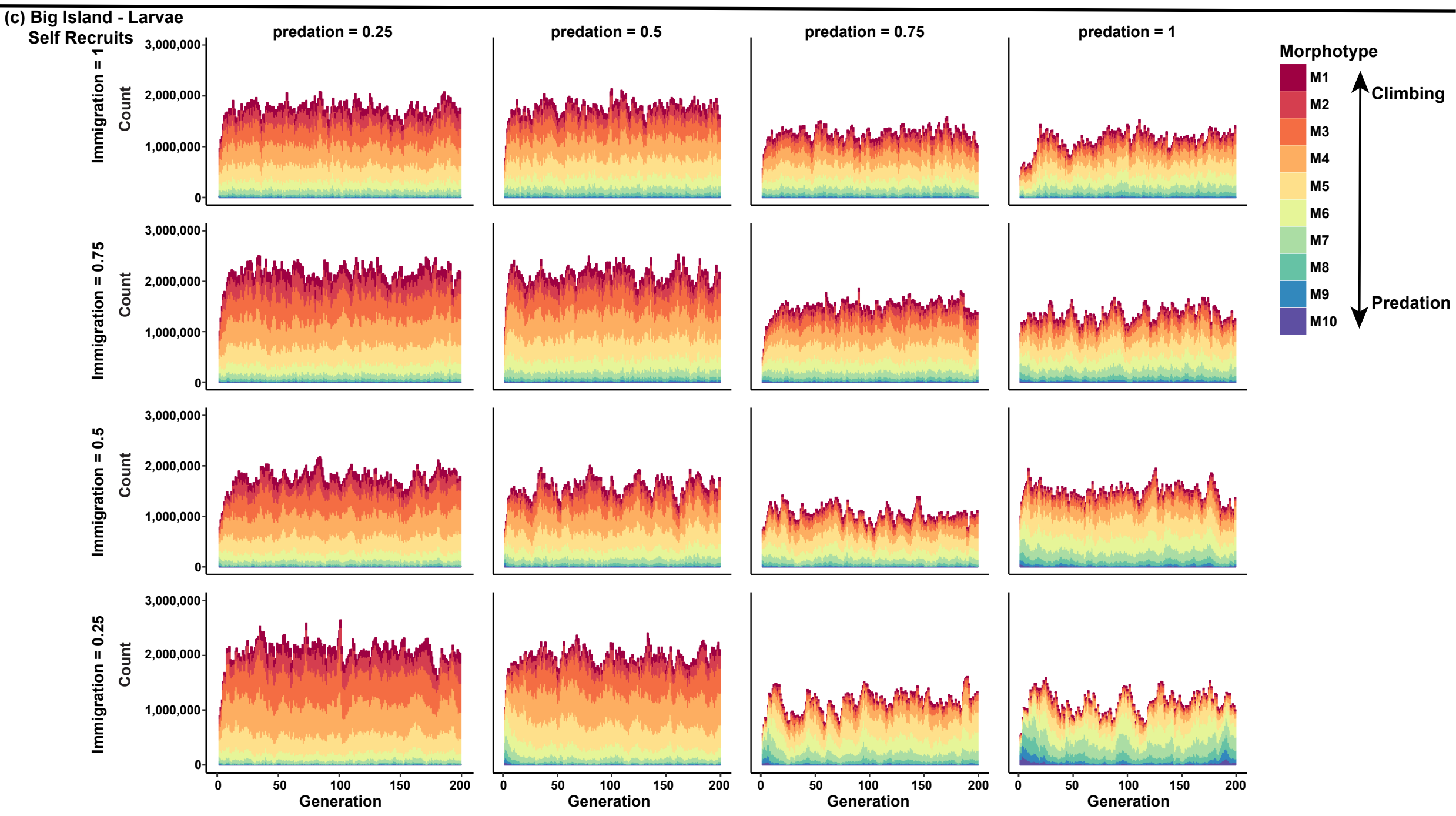

Supplement: Supplementary file 3 — Figure S3. Simulated counts of self-recruitment larval morphotypes for 200 generations on the islands of Kaua‘i (a), O‘ahu (b), and the Big Island (c) from the individual-based models of immigration (ranging from 25%-100%) with varying levels (0.25 to 1) of post-settlement predation selection (scenario 4). Warm colors represent climbing morphotypes (M1-M4) and cool colors represent predation evasion morphotypes (M7-M10). (PDF 4769 kb) [file 12862_2019_1413_MOESM3_ESM.pdf]
