# Supplementary material for: Evidence of local adaptation in a waterfall-climbing Hawaiian goby fish derived from coupled biophysical modeling of larval dispersal and post-settlement selection
Source: BMC Evol Biol. 2019 Apr 11;19:88. doi: 10.1186/s12862-019-1413-4 (PMC6458715; doi:10.1186/s12862-019-1413-4)
Supplement: Supplementary file 4 — Supplemental Equations. Sine curve equations of monthly stream discharge estimates from UGSS stream gauge data. (DOCX 101 kb) [file 12862_2019_1413_MOESM4_ESM.docx]

Additional File 1

Supplemental Equations - Sine curve equations of monthly stream discharge estimates from UGSS stream gauge data.

flow_H1_ = (0.196 + 1.00 * SIN((6.283 * week / 17 + 3.8) * 180 / pi))

flow_H2_ = (2.054 + 0.30 * SIN((6.283 * week / 17 + 3.8) * 180 / pi))

flow_H3_ = (1.939 + 0.30 * SIN((6.283 * week / 17 + 3.8) * 180 / pi))

flow_H4_ = (1.994 + 0.30 * SIN((6.283 * week / 17 + 3.8) * 180 / pi))

flow_H5_ = (1.299 + 0.30 * SIN((6.283 * week / 17 + 3.8) * 180 / pi))

flow_H6_ = (1.865 + 0.30 * SIN((6.283 * week / 17 + 3.8) * 180 / pi))

flow_H7_ = (1.345 + 0.40 * SIN((6.283 * week / 17 + 3.8) * 180 / pi))

flow_H8_ = (1.239 + 0.30 * SIN((6.283 * week / 17 + 3.8) * 180 / pi))

flow_H9_ = (1.860 + 0.18 * SIN((6.283 * week / 17 + 3.8) * 180 / pi))

flow_O1_ = (1.567 + 0.25 * SIN((6.283 * week / 51 + 1) * 180 / pi))

flow_O2_ = (0.995 + 0.25 * SIN((6.283 * week / 51 + 1) * 180 / pi))

flow_O3_ = (0.519 + 0.60 * SIN((6.283 * week / 51 + 1) * 180 / pi))

flow_O4_ = (1.075 + 0.50 * SIN((6.283 * week / 51 + 1) * 180 / pi))

flow_O5_ = (1.015 + 0.25 * SIN((6.283 * week / 51 + 1) * 180 / pi))

flow_O6_ = (1.554 + 0.15 * SIN((6.283 * week / 51 + 1) * 180 / pi))

flow_O7_ = (1.013 + 0.15 * SIN((6.283 * week / 51 + 1) * 180 / pi))

flow_O8_ = (0.958 + 0.15 * SIN((6.283 * week / 51 + 1) * 180 / pi))

flow_O9_ = (1.397 + 0.45 * SIN((6.283 * week / 51 + 1) * 180 / pi))

flow_K1_ = (1.963 + 0.50 * SIN((6.283 * week / 51 + 1) * 180 / pi))

flow_K2_ = (1.911 + 0.25 * SIN((6.283 * week / 51 + 1) * 180 / pi))

flow_K3_ = (0.809 + 0.55 * SIN((6.283 * week / 51 + 1) * 180 / pi))

flow_K4_ = (2.060 + 0.25 * SIN((6.283 * week / 51 + 1) * 180 / pi))

flow_K5_ = (0.364 + 0.25 * SIN((6.283 * week / 51 + 1) * 180 / pi))

flow_K6_ = (1.520 + 0.45 * SIN((6.283 * week / 51 + 1) * 180 / pi))

flow_K7_ = (1.520 + 0.25 * SIN((6.283 * week / 51 + 1) * 180 / pi))

flow_K8_ = (2.115 + 0.15 * SIN((6.283 * week / 51 + 1) * 180 / pi))

flow_K9_ = (1.203 + 0.30 * SIN((6.283 * week / 51 + 1) * 180 / pi))
