# Supplementary material for: Evidence of local adaptation in a waterfall-climbing Hawaiian goby fish derived from coupled biophysical modeling of larval dispersal and post-settlement selection
Source: BMC Evol Biol. 2019 Apr 11;19:88. doi: 10.1186/s12862-019-1413-4 (PMC6458715; doi:10.1186/s12862-019-1413-4)
Supplement: Supplementary file 5 — Figure S4. Predicted morphotype frequency distribution and island of origin of settled pelagic larvae from the dispersal. These frequency distributions were used as the inputs for pelagic larval morphology (Stage 1) in the individual-based models that included immigration (scenarios 3, and 4). (PDF 36 kb) [file 12862_2019_1413_MOESM5_ESM.pdf]

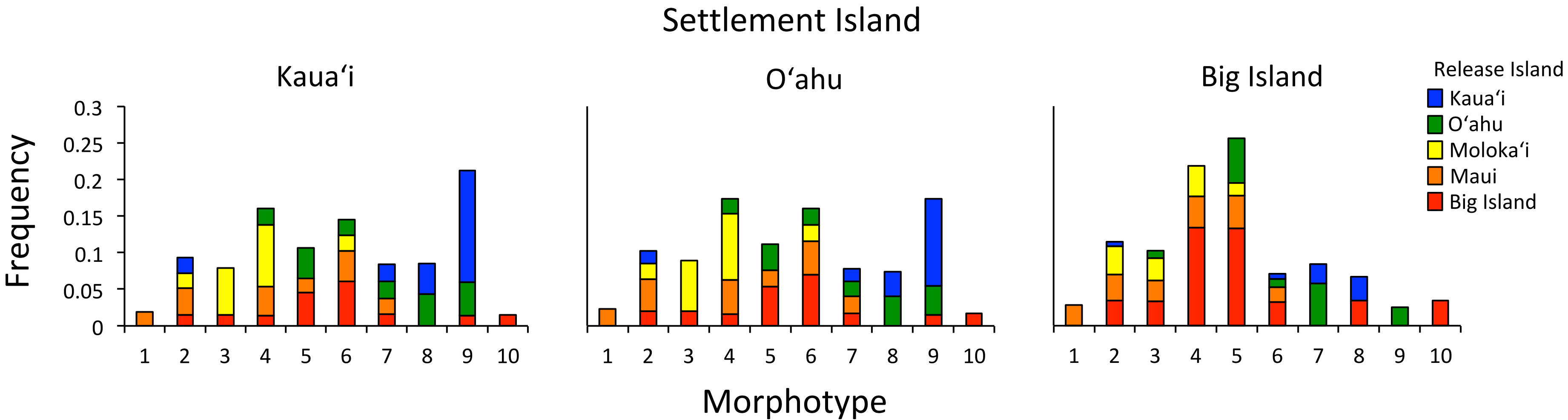
Settlement Island
Kaua'i
O'ahu
Big Island
Release Island
Kaua'i
O'ahu
Moloka'i
Maui
Big Island
Frequency
0.3
0.25
0.2
0.15
0.1
0.05
0
1 2 3 4 5 6 7 8 9 10
Morphotype
