# Supplementary material for: Evidence of local adaptation in a waterfall-climbing Hawaiian goby fish derived from coupled biophysical modeling of larval dispersal and post-settlement selection
Source: BMC Evol Biol. 2019 Apr 11;19:88. doi: 10.1186/s12862-019-1413-4 (PMC6458715; doi:10.1186/s12862-019-1413-4)
Supplement: Supplementary file 1 — Figure S1. Simulated counts of larval morphotypes for 200 generations on the islands of Kaua‘i (a), O‘ahu (b), and the Big Island (c) from the individuals-based models of isolation with varying levels (0 to 1) of post-settlement selection of predation (columns) and climbing (rows) (scenario 2). Warm colors represent climbing morphotypes (M1-M4) and cool colors represent predation evasion morphotypes (M7-M10). (PDF 4133 kb) [file 12862_2019_1413_MOESM1_ESM.pdf]

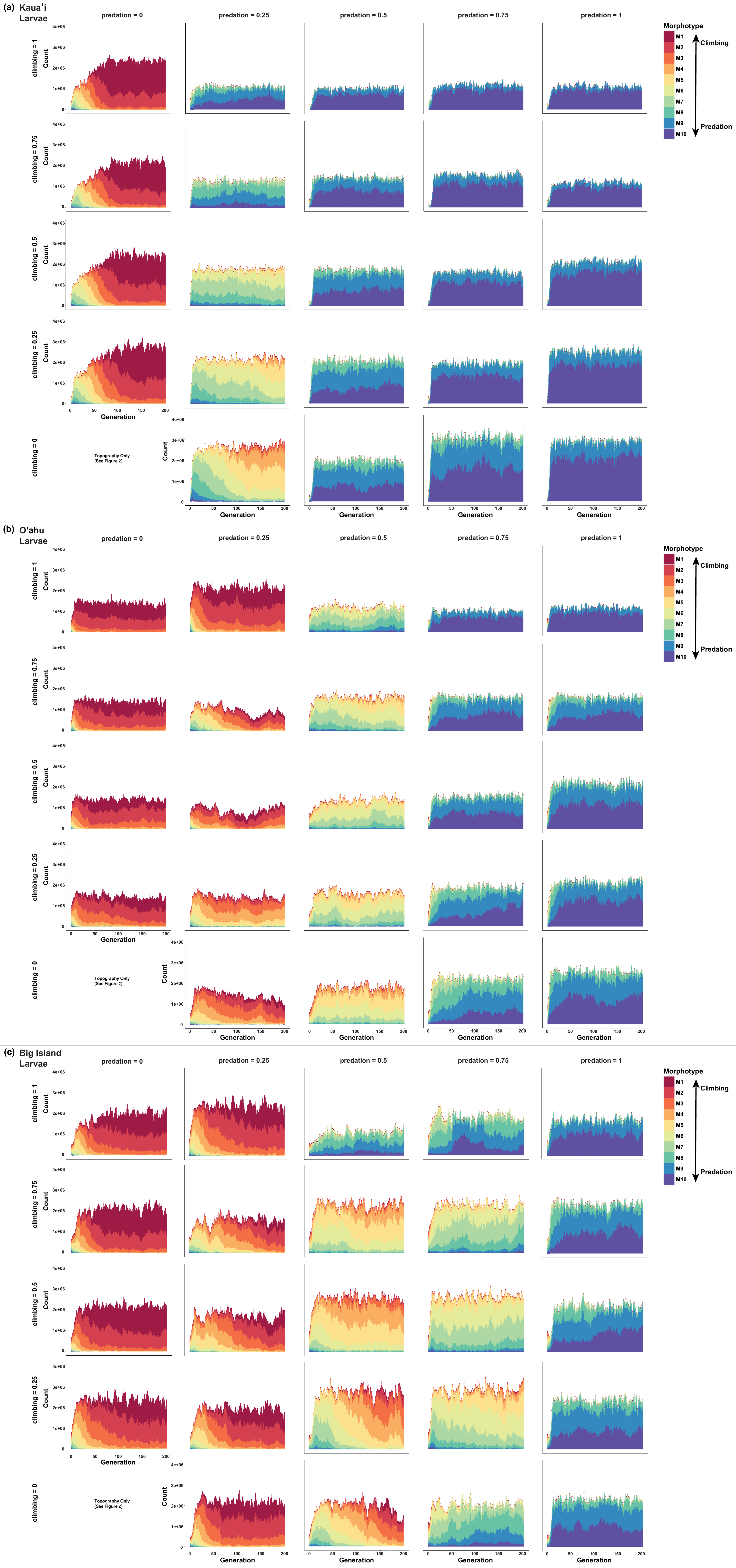

(a) Kauaʻi
Larvae
(b) Oʻahu
Larvae
(c) Big Island
Larvae
predation = 0
predation = 0.25
predation = 0.5
predation = 0.75
predation = 1
climbing = 1
climbing = 0.75
climbing = 0.5
climbing = 0.25
climbing = 0
Count
Generation
Topography Only
(See Figure 2)
Morphotype
M1
M2
M3
M4
M5
M6
M7
M8
M9
M10
Climbing
Predation
